# RNAelem: an algorithm for discovering sequence-structure motifs in RNA bound by RNA-binding proteins

**DOI:** 10.1093/bioadv/vbae144

**Published:** 2024-09-28

**Authors:** Hiroshi Miyake, Risa Karakida Kawaguchi, Hisanori Kiryu

**Affiliations:** Department of Computational Biology and Medical Sciences, Graduate School of Frontier Sciences, University of Tokyo, Chiba 277-8561, Japan; Department of Life Science Frontiers, Center for iPS Cell Research and Application (CiRA), Kyoto University, Sakyo-ku 606-8507, Japan; Department of Computational Biology and Medical Sciences, Graduate School of Frontier Sciences, University of Tokyo, Chiba 277-8561, Japan

## Abstract

**Motivation:**

RNA-binding proteins (RBPs) play a crucial role in the post-transcriptional regulation of RNA. Given their importance, analyzing the specific RNA patterns recognized by RBPs has become a significant research focus in bioinformatics. Deep Neural Networks have enhanced the accuracy of prediction for RBP-binding sites, yet understanding the structural basis of RBP-binding specificity from these models is challenging due to their limited interpretability. To address this, we developed RNAelem, which combines profile context-free grammar and the Turner energy model for RNA secondary structure to predict sequence-structure motifs in RBP-binding regions.

**Results:**

RNAelem exhibited superior detection accuracy compared to existing tools for RNA sequences with structural motifs. Upon applying RNAelem to the eCLIP database, we were not only able to reproduce many known primary sequence motifs in the absence of secondary structures, but also discovered many secondary structural motifs that contained sequence-nonspecific insertion regions. Furthermore, the high interpretability of RNAelem yielded insightful findings such as long-range base-pairing interactions in the binding region of the U2AF protein.

**Availability and implementation:**

The code is available at https://github.com/iyak/RNAelem.

## 1 Introduction

RNA-binding proteins (RBPs) play a pivotal role in post-transcriptional regulation, orchestrating a variety of biological processes such as splicing, transport, localization, translation, and degradation of RNA (Ray *et al.* 2013, Gerstberger *et al.* 2014). A central question in understanding the functions of RBPs is how they recognize and bind specific sites on RNA molecules, requiring investigation of the primary sequence and secondary structural features around the RBP binding sites.

Cross-linking and immunoprecipitation sequencing (CLIP-Seq) is a potent tool for delineating interactions between RBPs and RNA on a genomic scale (Yeo *et al.* 2009, Hafner *et al.* 2010, König *et al.* 2010, Van Nostrand *et al.* 2016). However, the binding regions identified by CLIP-Seq are usually hundreds of base pairs long, posing a challenge in pinpointing the exact areas of physical contact between RBPs and RNA. Furthermore, CLIP-Seq results depend on cell type and culture conditions, potentially precluding the detection of binding regions in RNAs with low or no expression in the sampled cells (Bottini *et al.* 2018). Thus, bioinformatics analysis to infer RBP-binding regions and motif sequences from CLIP-Seq data is indispensable.

Deep neural network (DNN) technologies have significantly enhanced the precision of distinguishing CLIP-Seq peak regions from background sequences. iDeepS uses convolutional neural networks (CNN) to encode the primary sequence and predicted secondary structure, subsequently processed by bidirectional long short-term memory networks to capture long-span feature distributions (Pan *et al.* 2018). GraphProt2 employs graph convolution to extract features from secondary sub-structures (Uhl *et al.* 2021). BERT-RBP leverages a pre-trained BERT model, fine-tuned for RBP binding prediction tasks, thereby harnessing hidden features such as transcript domain types and higher-order structures (Yamada and Hamada 2022).

While these tools significantly enhance the accuracy of identifying RBP-binding regions and primary sequence motifs within CLIP-Seq peak regions, typical DNN models contain a large number of parameters for their high expressive power, complicating the interpretation of the trained models (Li *et al.* 2020). For instance, it is often unclear whether they are truly capturing the biological signals of RBP-binding regions or merely exploiting systematic biases in the background sequences used for training. Additionally, current DNN models struggle to identify RNA folding shapes of structural motifs in binding regions. Therefore, developing interpretable models that can detect common RNA folding shapes around RBP-binding regions from CLIP-Seq data is crucial.

Traditionally, bioinformatic analysis of RNA secondary structure has often been conducted using Zuker’s algorithm with Turner’s energy parameters (Mathews *et al.* 1999) or its essentially equivalent context-free grammar (CFG) algorithms (Kiryu *et al.* 2008). These algorithms enable the computation of minimum free energy structures or a summation of Boltzmann factors across an exponential number of possible secondary structures with respect to the sequence length in polynomial time. Various tools, including CMfinder, RNApromo, and GraphProt, utilize these algorithms to predict common structural motifs from multiple RNA sequences (Yao *et al.* 2006, Rabani *et al.* 2008, Maticzka *et al.* 2014).

CMfinder builds an initial Profile CFG model from sequence alignments, then iteratively refines it using the Expectation-Maximization algorithm (Yao *et al.* 2006). It employs base pairing probabilities computed by the energy model to detect more stable structural motifs. RNApromo first samples secondary structures for input RNA sequences and then learns a Profile CFG to identify base pair profiles and stem structure patterns (Rabani *et al.* 2008). GraphProt, designed for CLIP-Seq analysis, uses a graph kernel to extract characteristic features of sampled secondary structures and a support vector machine (SVM) to learn feature weights (Maticzka *et al.* 2014). Although GraphProt lacks internal motif models, it can output the base frequency and structure profile of predicted binding sites through post-analysis.

**Figure 1.**
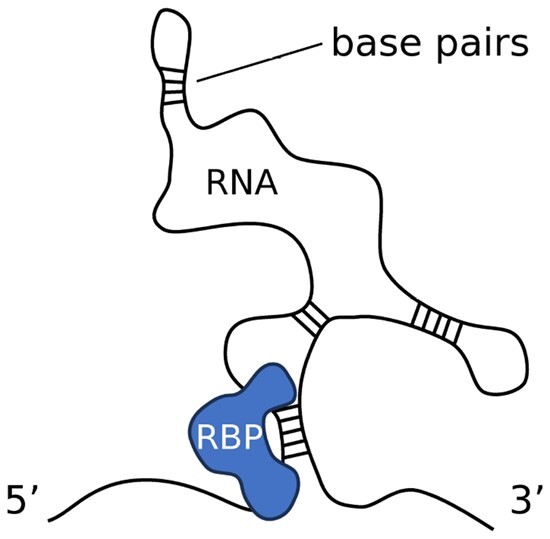
A possible molecular configuration of RBP-binding regions. In this configuration, RBP binds to the RNA region containing base pairs, but it is insensitive to the long insertion region (upper part).

However, from our perspective, these structural models are unsatisfactory for RBP-binding motif search. While CMFinder and RNApromo were originally developed to classify structural RNA families and thus only consider contiguous RNA regions, an RBP might recognize and bind to a small stem region, remaining insensitive to the sequence enclosed by the base pairs (Corley *et al.* 2020; Fig. 1). An example is the double-stranded RNA-binding protein STAU1, which regulates mRNA localization, translation, and decay. One of the STAU1-binding regions is the stem region of the ARF1 gene’s 3′UTR, interspersed with a long insertion region of approximately 100 nucleotides. Yadav *et al.* demonstrated that STAU1 does not exhibit binding specificity to the insertion region by showing that STAU1 binds to a dsRNA, which is obtained by removing the insertion region from the binding RNA region (Yadav *et al.* 2020) (PDB ID: 6SDY). In such cases, the structural motif should consist of multiple RNA sequence regions that are distant at the primary sequence level yet spatially proximal to the bound RBP.

GraphProt, on the other hand, provides only base-wise structure profiles, such as stem probability and loop probabilities at each base position, without identifying position pairs that form hydrogen bonds. This limitation significantly constrains downstream analysis and validation of structural motifs.

To address these issues, we developed RNAelem, an algorithm that predicts sequence-structure motifs in RBP-binding regions with base-pair resolution. RNAelem can identify motifs that allow long-range, structure-unspecified insertions from sequences of CLIP-Seq peak regions. Additionally, we devised a highly interpretable visualization method for sequence-structure motifs, extending the sequence logo concept to display positional relationships between secondary structures and base profiles. This method also incorporates base-pair logos to visualize the specificity of base pair frequencies.

Experiments with simulated data confirm that RNAelem surpasses existing tools in detecting RNA sequences containing structural motifs. In tests with actual CLIP-Seq data, RNAelem markedly outperforms tools that generate secondary structural motifs in distinguishing between CLIP-seq peak and background sequences, although it falls short of tools that use DNN and SVM and do not output base-paring partners. Leveraging the interpretability of the RNAelem model, we analyzed sequence-structure motifs in RBP-binding regions using data from the eCLIP database (Van Nostrand *et al.* 2016). This analysis revealed intriguing results, including the identification of novel structural motifs spanning long sequence regions.

## 2 Methods

### 2.1 Core concept of RNAelem’s motif model

We have developed a novel motif model based on a Coupled Context-Free Grammar (CFG), integrating two distinct CFGs: Profile CFG and CFG of the Turner energy model for secondary structures. This section first outlines the design concept of the Coupled CFG before detailing its implementation.

To represent the RBP-binding preferences at both base and base-pair levels, including the situation in Fig. 1, we utilize a Profile CFG similar to Infernal (Eddy 2002, Nawrocki and Eddy 2013). Each parse tree ϕ, a tree-structured sequence of hidden state transitions, of the Profile CFG segments an RNA sequence into motif matching regions and other background regions. Accurately fitted parameters θ of the Profile CFG increase the likelihood of parse tree ϕ that identifies the RBP binding site. However, the Profile CFG alone often results in many false matches, as RBP-binding regions are typically only a few bases in length. This makes it difficult to learn the parameters θ of the Profile CFG from RNA sequences effectively.

To mitigate this issue, we consider learning the parameters from only the matching regions that have a stable structure consistent with the base-pairing pattern of the Profile CFG. Specifically, we consider a Coupled CFG which combines Profile CFG and CFG of the Turner energy model, and define a scoring function that evaluates both aspects.

Our Profile CFG’s grammar is encoded by a string called a search pattern μ, akin to the dot-bracket notation used for RNA secondary structures (refer to the “Definition of search pattern” section for details). Let $x\in {\left\{A,C,G,U\right\}}^{L}$ be an RNA sequence of length *L*, and let ϕ and σ be parse trees of the Profile CFG and the energy model for *x*, respectively. Additionally, let τ be the parse tree of the Coupled CFG associated with ϕ and σ. The likelihood $P_{\mathrm{couple}}$ of the parse tree τ is defined by:


(1) $$P_{\mathrm{couple}}(\tau |\mu ,\theta ,\lambda ,x)=\frac{{\tilde{P}}_{\mathrm{energy}}{\left(\sigma |x\right)}^{\lambda }{\tilde{P}}_{\mathrm{profile}}(\phi |\mu ,\theta ,x)}{Z(\mu ,\theta ,\lambda ,x)}$$

where ${\tilde{P}}_{\mathrm{profile}}$ and ${\tilde{P}}_{\mathrm{energy}}$ represent the unnormalized probabilities of each parse tree; *Z* the partition function; θ the parameters of the Profile CFG; and λ a trainable parameter that balances the relative strengths between motif match and energetic stability.

As shown in Equation (1), we multiply the unnormalized probability of the Profile CFG by that of the energy model. The latter factor acts as a variable weight for each matching position in the training sequence. This means that matching regions where the base-pairing structure is consistent between the Profile CFG and the energy model receive higher weights during the training process. This approach is designed to address the false matching problem and enhance the precision of the motif model.

### 2.2 Procedures from input to output

**Figure 2.**
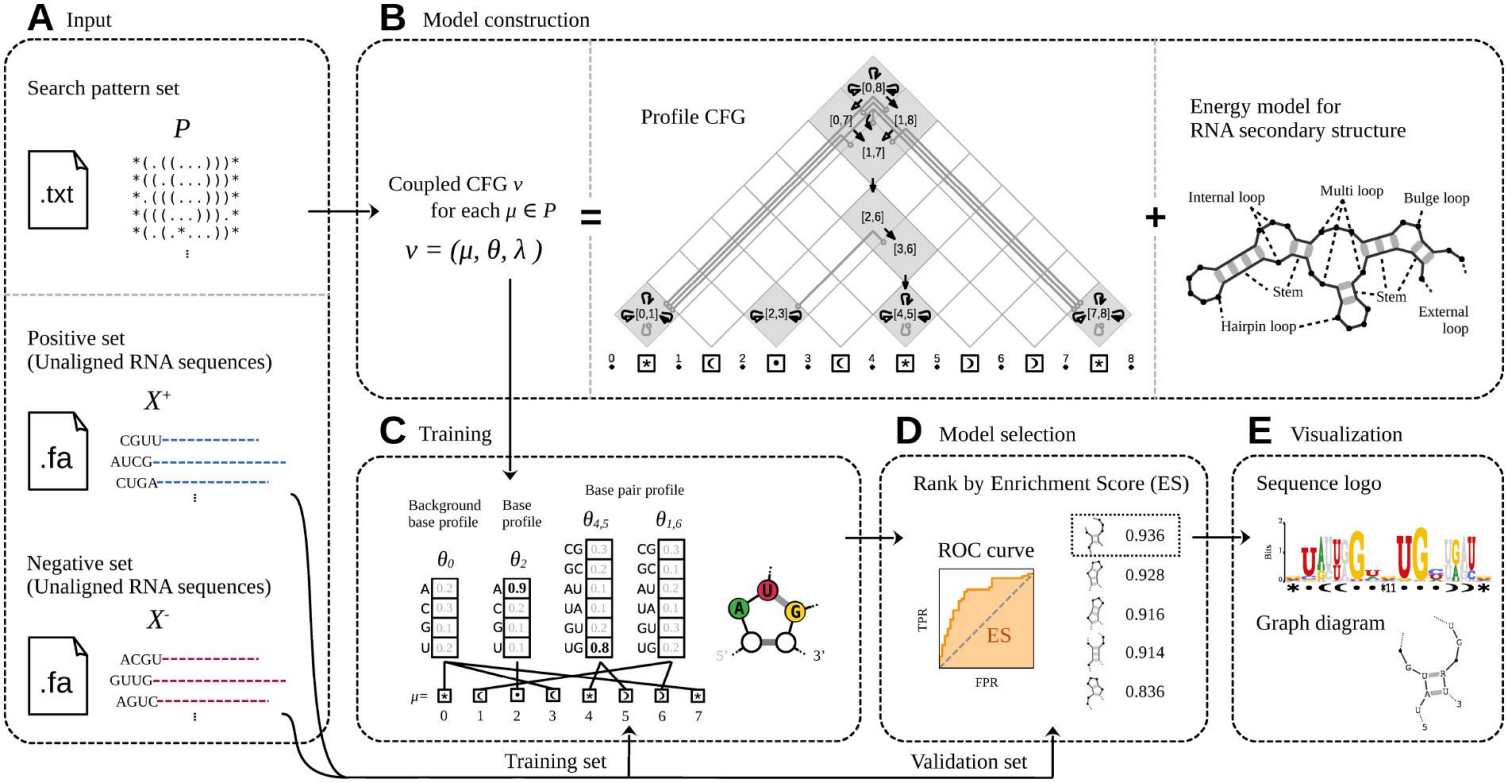
Flow diagram of motif discovery process using RNAelem. The motif discovery process in RNAelem involves: (A) inputting both a positive and negative RNA sequence set, and a search pattern set; (B) developing a Coupled Context-Free Grammar (CFG) for each pattern, merging Profile CFG with an Energy model to balance strengths via parameter λ, illustrated by a Profile CFG example; (C) training Coupled CFG parameters using a selected input sequence set; (D) computing and ranking each CFG’s Enrichment Score (ES) using a validation set; (E) identifying and visualizing the highest-scoring CFG as the best motif.

In the training phase, the inputs are a set of positive sequences $X^{+}=\{x_{1}^{+},\ldots ,x_{N^{+}}^{+}\}$, a set of negative sequences $X^{-}=\{x_{1}^{-},\ldots ,x_{N^{-}}^{-}\}$, and a set P of search patterns (Fig. 2A). Positive sequences $X^{+}$ typically consist of unaligned RNA sequences from CLIP-seq data peak regions, while negative sequences $X^{-}$ are sampled from non-peak regions or are artificially shuffled sequences. The search pattern set P, which defines the search space of structural motifs, is by default generated internally using Algorithm but can also be user-supplied. For this research, we employed a predefined set of 135 search patterns, as listed in Supplementary Table S1.

RNAelem begins by transforming each search pattern μ∈P into a corresponding Coupled CFG ν=(μ,θ,λ), where θ is the base/base-pair profile and λ is the scaling factor, to form the Coupled CFG set N (Fig. 2B). Details of this transformation are in the “Construction of Coupled CFG” section. A *K*-fold cross-validation approach is employed to iteratively train each Coupled CFG ν∈N, where we partition the data into training sets $X_{k}^{+}$ and $X_{k}^{-}$ for each *k*-th fold and fit ν to these subsets (Fig. 2C). The training process is further elaborated in the “Training of Coupled CFG” section.

For each Coupled CFG ${\hat{\nu }}_{k}=(\mu ,{\hat{\theta }}_{k},{\hat{\lambda }}_{k})$, where ${\hat{\theta }}_{k}$ and ${\hat{\lambda }}_{k}$ are the trained parameters in the *k*-th round of cross-validation, the Enrichment Score (ES) is calculated to evaluate the significance of the motif model (Fig. 2D). ES, defined as the Area Under the Receiver Operating Characteristic curve (AUROC), measures the accuracy of discriminating between the positive and negative subsets of sequences not used for training. Further details on this computation are provided in the Supplementary “Computing Enrichment Score (ES)” section and Algorithm S4.

The Coupled CFGs ν∈N are ranked based on their ES (Fig. 2C). We then select the Coupled CFG $\nu_{\max}$ with the highest ES for a final round of training using the complete sets of $X^{+}$ and $X^{-}$. This training refines its parameters. RNAelem outputs the final motif model ${\hat{\nu }}_{\text{max}}$, accompanied by its corresponding ES (Fig. 2E).

Supplementary Algorithm S5 shows the entire workflow for training and selecting the motif model.

### 2.3 Definition of search pattern

To delineate the search space for structural motifs reflecting most binding configurations of RBPs, we introduce the search pattern μ. This pattern is defined using a modified dot-bracket notation for secondary structures. In addition to the standard characters “(”, “)”, and “.”, a wildcard character “*” is employed to match any length of structure. Each dot and bracket symbolizes a segment of the RBP-binding motif with specific base and base-pair frequency profiles θ. Brackets must be balanced and free of pseudoknots, akin to ordinary secondary structures. The motif must begin and end with “*” to enable pattern matching at any sequence position. For instance, the pattern “*(.(*))*” depicts a bulge loop motif consisting of two stem regions that enclose an unpaired region, with the middle insertion region accommodating any structure within the span of the internal bracket.

The set of search patterns P defines the search space. A default of 135 search patterns is systematically selected, as detailed in the supplementary section “Candidate Patterns and Model Selection.” This strategy is designed to cover the theoretical secondary structure space as comprehensively as possible while avoiding computational explosion. This approach aims to discover novel structural motifs that have been unidentified.

### 2.4 Construction of coupled CFG

For each search pattern μ, RNAelem first constructs a Profile CFG model (Fig. 2B and Supplementary Fig. S1). Initially, we allocate log-scaled scores for two types of emissions in the model: Base emissions and base pair emissions. These include $\theta_{0}$ for insertion regions, $\theta_{i}$ for dots, and $\theta_{i,j}$ for bracket pairs as shown in Fig. 2C. We then allocate hidden states for dynamic programming, corresponding to the intervals in search pattern μ (Fig. 2B and Supplementary Algorithm S1). Then, a transition graph is constructed between these hidden states (Supplementary Algorithm S2). To accommodate variations in stem or loop length within motif regions, each state allows self-transitions with a constant minor weight ω, also log-scaled.

On the other hand, state deletions are not included in our profile CFG models, as they would reduce both the specificity of sequence matching and the interpretability of the resulting motifs. Allowing deletions could cause a motif to match various RNA structural topologies, leading to ambiguity in the optimization function. Furthermore, visualizing motifs with deletions would complicate interpretation. Therefore, we concluded that the disadvantages of allowing deletions outweigh any potential benefits and chose not to include them in the model. For a more detailed discussion, refer to the Supplementary section “Comparison with Infernal’s Profile CFG”.

Now, we define the matching score of the Profile CFG. The unnormalized probability of a parse tree ϕ, representing the tree-structured state transitions of a Profile CFG for sequence *x*, is defined as Equation (2):


(2) $$\begin{array}{c} {\tilde{P}}_{\mathrm{profile}}(\phi |\mu ,\theta ,x)=\text{exp}(\sum_{\begin{array}{c} i\in H_{\mathrm{loop}},\\ a\in B_{1} \end{array}}\theta_{i,a}N_{i,a}(\phi ,x)\\ \;\;\;\;+\sum_{\begin{array}{c} (i,j)\in H_{\mathrm{pair}},\\ (b_{1},b_{2})\in B_{2} \end{array}}\theta_{i,j,b_{1},b_{2}}N_{i,j,b_{1},b_{2}}(\phi ,x)\\ +\sum_{a\in B_{1}}\theta_{0,a}N_{0,a}(\phi ,x)+\omega N(\phi ,x)) \end{array}$$

where $H_{\mathrm{loop}}$ is the index set for profile θ used in base emissions by loop states, and $H_{\mathrm{pair}}$ is used in base-pair emissions by stem regions. $B_{1}$ and $B_{2}$ represent all bases {A,C,G,U} and base pairs {AU,CG,GC,GU,UA,UG}, respectively. $N_{i,a}(\phi ,x)$ and $N_{i,j,b_{1},b_{2}}(\phi ,x)$ count the emissions of base *a* by symbol $\mu_{i}$ and base pairs $b_{1},b_{2}$ by bracket symbols $\mu_{i},\mu_{j}$, respectively. $N_{0,a}(\phi ,x)$ counts the base emissions from the insertion regions, and N(ϕ,x) is the total number of self-transitions in ϕ.

Subsequently, we define the other component of the Coupled CFG, RNA secondary structure energy model, specifically the Turner energy model (Mathews *et al.* 1999). We used the CFG from the reference (Kiryu *et al.* 2008), which constrains the maximal span *W* between pair-forming bases. Let σ be a parse tree of the energy model, which represents one of potential secondary structures of sequence *x*. The unnormalized probability ${\tilde{P}}_{\mathrm{energy}}$ of parse tree σ for sequence *x* is given by Equation (3):


(3) $${\tilde{P}}_{\mathrm{energy}}(\sigma |x)=\text{exp}\;\sum_{p\in \sigma }-\frac{1}{kT}\Delta G(p,x)$$

where *k* is Boltzmann’s constant; *T* temperature; and ΔG the free energy change associated with state transition *p*.

In our method, we integrate these two CFGs. The parse tree for the Coupled CFG is denoted as τ=σ⊗ϕ, where ⊗ indicates the alignment of base and base-pair emissions in σ and ϕ, ensuring that τ decomposes into σ and ϕ with consistent emissions. The probability of parse tree τ is calculated using Equation (1), where the partition function Z(ν,x) is defined as:


(4) $$Z(\nu ,x)=\sum_{\tau \in \Phi (x)}{\tilde{P}}_{\mathrm{couple}}(\tau |\nu ,x)$$

where Φ(x) represents all possible parse trees for the sequence *x*. This coupling technique allows for the calculation of the joint distribution of search pattern matching and secondary structural formation.

For parameter training, we employ a Conditional Random Field (CRF) (Lafferty *et al.* 2001). Let $\Phi^{+}(x)$ represent the set of parse trees such that a full-length motif occurs at some position in sequence *x*. Defining the event of the motif’s presence in sequence *x* as y=1 and its absence as y=0, the probability of the motif’s occurrence is given by Equation (5):


(5) $$P(y=1|\nu ,x)=\frac{Z^{+}(\nu ,x)}{Z(\nu ,x)},$$

(6) $$\begin{array}{cc} Z^{+}(\nu ,x) & =\sum_{\tau \in \Phi^{+}(x)}{\tilde{P}}_{\mathrm{couple}}(\tau |\nu ,x) \end{array}$$

Conversely, the probability of motif’s absence in *x*, P(y=0|ν,x), is similarly formalized as $Z^{-}(\nu ,x)/Z(\nu ,x)$.

### 2.5 Training of coupled CFG

RNAelem optimizes parameters to maximize the difference in likelihood between the positive set $X^{+}$ and the negative set $X^{-}$ using stochastic gradient descent. The objective function is defined as Equation (7):


(7) $$L(X^{+},X^{-},\nu )=\sum_{n=1}^{N_{+}}\;\text{log}\;P(y_{n}=1|\nu ,x_{n}^{+})+\sum_{n=1}^{N_{-}}\;\text{log}\;P(y_{n}=0|\nu ,x_{n}^{-})-R(\theta ,\lambda )$$

where R(θ,λ) represents a regularization term.

The objective function is differentiable with respect to all parameters θ and λ. For example, the differentiation by the base profile $\theta_{i,a}$ is calculated as Equation (8):


(8) $$\begin{array}{c} \frac{\partial }{\partial \theta_{i,a}}L(X^{+},X^{-},\nu )=\sum_{n=1}^{N^{+}}E^{+}\left[N_{i,a}(\phi ,x_{n}^{+})\right]-E\left[N_{i,a}(\phi ,x_{n}^{+})\right]\\ +\sum_{n=1}^{N^{-}}E^{-}\left[N_{i,a}(\phi ,x_{n}^{-})\right]-E\left[N_{i,a}(\phi ,x_{n}^{-})\right]\\ +\frac{\partial }{\partial \theta_{i,a}}R(\theta ,\lambda ) \end{array}$$

where $E^{+}$, $E^{-}$ and *E* represent the expected values associated with partition functions $Z^{+}$, $Z^{-}$ and *Z*, respectively. These expected values across all possible secondary structures and motif occurrence patterns are computed using a dynamic programming inspired by Sankoff’s method (Sankoff 1985), called inside and outside algorithms (Supplementary Fig. S2).

In the Supplementary Methods, we provide a detailed description of each step, an analysis of time complexity, and execution time (Supplementary Table S5). Additionally, to mitigate the challenges of overfitting, local optima, and dependency on initial values during optimization, several strategies are delineated, including a novel method termed “simulated negative,” which significantly enhances performance (Supplementary Fig. S3).

### 2.6 Visualization of sequence-structure motif

**Figure 3.**
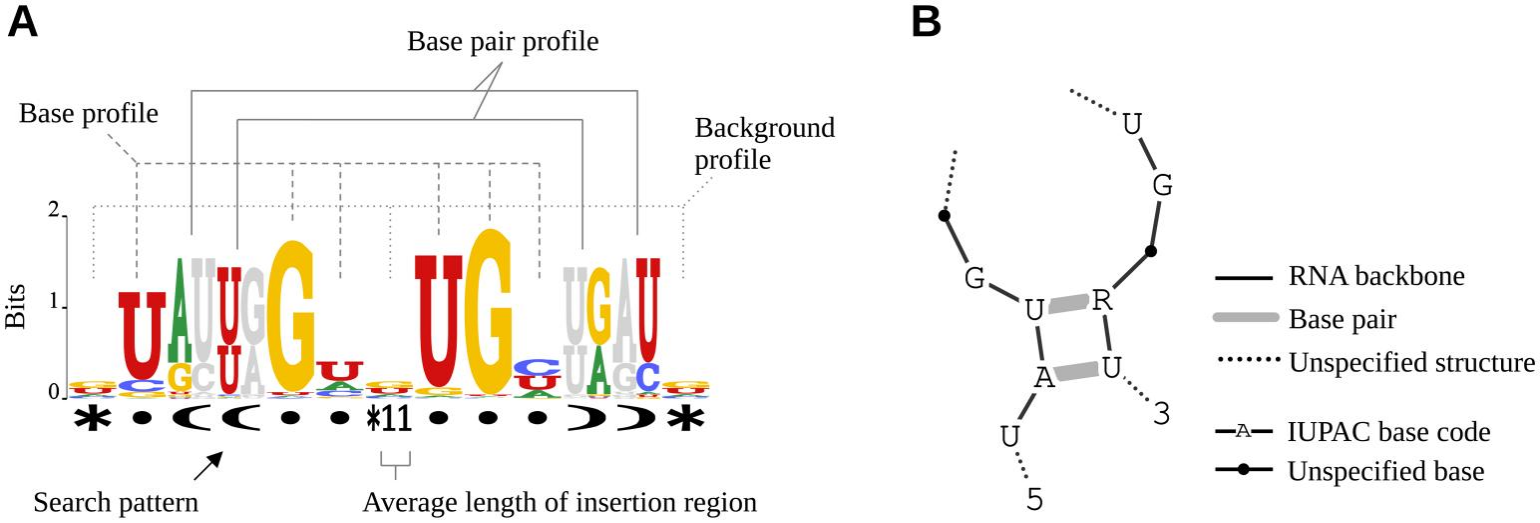
Secondary structural motifs output by RNAelem. The CLIP-Seq results from ELAC2 are utilized to showcase the two types of output. (A) Sequence logos, customized for sequence-structure motif, displaying information content in bits across single-stranded and stem regions, with insertion regions highlighted by average lengths. (B) Graphical diagrams that amalgamate search patterns and profiles for enhanced interpretability, emphasizing conserved sequences with IUPAC base codes in bases where informational content is greater than 1.5 bits.

The motif models with trained parameters are visualized to enhance interpretability. Figure 3 illustrates a sequence-structure motif generated by RNAelem, displayed as an extended sequence logo and a graph diagram. We extend the sequence logo, which typically shows a primary sequence motif, by displaying the search pattern μ below the logo and incorporating base pair proﬁles in pairing regions. The profile height represents its information content, peaking at ${\;\text{log}\;}_{2}(4)$ for single-stranded areas and ${\;\text{log}\;}_{2}(6)$ for paired regions. Information content is derived by exponentiating and column-normalizing the log-scaled profile parameters θ. Bases of pairing partners are shown in gray. A common background profile is shown in all insertion regions. The average gap length in the search pattern μ is indicated, except at the beginning and end regions. Thus, the motif logo generated by RNAelem not only visualizes the predicted motifs but also comprehensively represents the model structure of the learned profile CFG.

The graph diagram provides a visual interpretation of the sequence and secondary structure, with the backbone in black lines, hydrogen bonds in gray, and dotted lines for insertions. Conserved bases in columns with information content above 1.5 are marked with IUPAC codes, offering a comprehensive visualization of sequence-structure motifs.

### 2.7 Data preparation

In this study, we use both simulated and biological datasets. Due to the absence of a large-scale experimental database on secondary structure motifs in RBP-binding regions and limited knowledge about the distribution of these motifs in secondary structure space, we extracted structures uniformly from the entire secondary structure space as the true motif secondary structure for the simulated dataset.

We employed Supplementary Algorithm S3 to systematically enumerate the secondary structures. We then created simulated sequences embedding sequence-structure motifs using antaRNA, which outputs RNA sequences based on a given target secondary structure, focusing on local structures (Kleinkauf *et al.* 2015). This process generated 200 positive sequences for each of 43 structural motifs from local secondary structures of length 10, with gaps of length ranging from 1 to 10. The Vienna RNA package (Lorenz *et al.* 2011) ensured these sequences folded into the expected structure. All motifs are listed in Supplementary Table S2, and their conservation levels are depicted in Supplementary Fig. S4. Negative sequences were generated without specifying a target structure. A decoy set was created by randomly relocating motifs within sequences, thus disrupting the structure without altering sequence composition, to test the model’s ability to recognize both sequence and structure.

Biological sequences were obtained from eCLIP-Seq (Van Nostrand *et al.* 2016) data via the ENCODE project, covering 167 human RBP-binding proteins. Data were filtered to include regions with multiple replicates, using the top-1000 median peak scores for curation. Sequences shorter than 100 nucleotides were extended to ensure accurate secondary structure assessment. Datasets were divided equally for training and validation, with negative sequences sourced from non-peak regions of transcripts with at least one peak. Detailed data preparation methods are described in the Supplementary “Data preparation” section.

## 3 Results and discussion

### 3.1 Comparison of accuracy between tools using simulation data

**Figure 4.**
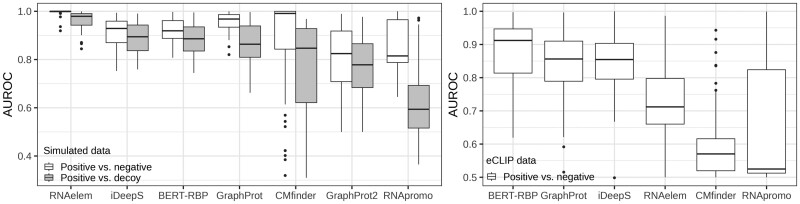
AUROC comparison across simulated and eCLIP data for various tools. AUROC values for 43 simulated datasets and 167 eCLIP datasets. The simulations involve discrimination between positive vs. negative, as well as positive vs. decoy. The box plot’s median is indicated by the central line, with the lower and upper hinges representing the 25th and 75th percentiles, respectively. Whiskers extend to the furthest values within 1.5 times the inter-quartile range from the hinges. Points beyond the whiskers are plotted individually.

We validated motif detection accuracy using a simulated dataset. Figure 4 and Supplementary Table S3 display sequence-wise AUROC accuracies for each tool. These tools were trained on positive and negative datasets to discriminate between another pair of positive and negative datasets, as well as positive and decoy datasets.

RNAelem achieved the highest mean AUROCs in both positive vs. negative and positive vs. decoy tasks (0.995/0.959). iDeepS (0.912/0.888) and BERT-RBP (0.919/0.880) posted comparable results, demonstrating the strong performance of DNN models that utilize convolution and transformer technologies. Conversely, GraphProt (0.954/0.865) and CMfinder (0.864/0.769) experienced significant decreases in their positive vs. decoy tasks, indicating that secondary structure conservation is often overlooked in the presence of sequence conservation. The scores for GraphProt2 (0.808/0.766), CMfinder (0.864/0.769), and RNApromo (0.861/0.632) varied significantly, suggesting that the performance of these tools heavily depends on the types of motif patterns being analyzed. RNApromo, originally developed for classifying RNA families, performed poorly in detecting RBP-binding motifs.

**Figure 5.**
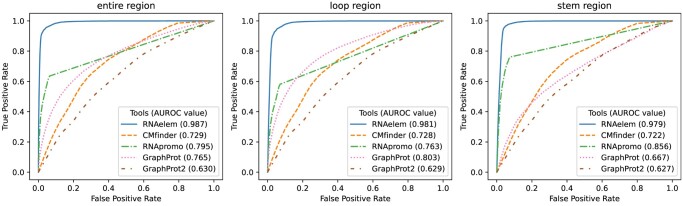
AUROC comparison for motif position estimation. The ROC curves illustrate the performance of each tool in discriminating whether each position belongs to a motif region. ROC curves were calculated separately for the entire motif region, the loop region, and the stem region.

We assessed the accuracy of motif position prediction across various tools, excluding iDeepS and BERT-RBP since they do not provide a motif presence score per position. By calculating the AUROC values using positive, negative, and decoy test sets, we evaluated the precision of motif region estimation (Figure 5 and Supplementary Table S4). Separate AUROC calculations for stem and loop regions underscored the impact of secondary structure context on accuracy. RNAelem demonstrated superior accuracy in pinpointing motif positions at single nucleotide resolution, indicating precise structural annotations in these areas. GraphProt exhibited significantly higher accuracy in loop regions compared to stem regions, suggesting less emphasis on stem region conservation. Conversely, RNApromo, a CFG-based method, excelled in stem region performance, reflecting its proficiency in identifying base pairing palindromes. However, GraphProt2, a deep learning approach, underperformed in motif position accuracy, likely due to its focus on global features from the sequence, as evidenced by its average prediction length of 38, which exceeds the accurate length of 12.

To accurately predict the motif position, it is crucial to segment sequences into conserved and background regions in terms of both primary sequence and secondary structure. While SVM/DNN models learn implicit features within sequences, RNAelem explicitly learns the profile on the search pattern, enabling precise estimation of conserved regions without surplus or deficit. Additionally, RNAelem minimizes false positives by aligning insertion regions with background areas.

Further analysis revealed notable variations in each tool’s performance based on the information content within the motif’s primary sequence (Supplementary Fig. S5). Additionally, the accuracy of RNAelem’s secondary structure predictions and the impact of maximum span *W* were validated by Matthews Correlation Coefficient scores, which compared predicted structures to ground truth (GT) in the motif regions (Supplementary Fig. S6). ES-based model selection also proved effective in reducing false positive motif detection (Supplementary Fig. S7). Furthermore, the selected search pattern differed from GT secondary structures, indicating that multiple search patterns can match the GT structure. This allows the best search pattern size to be chosen for precise GT motif alignment (Supplementary Fig. S8).

### 3.2 Validation of the accuracy of RNAelem on eCLIP data

Following confirmation of RNAelem’s accuracy with simulated data, we evaluated its performance on real eCLIP data. Since the true structural motifs in real data are unknown, we focused solely on comparing binding prediction accuracy. GraphProt2 was excluded from the comparison due to its unstable behavior in this dataset and our computing environment (Intel Core i9, GeForce RTX 2070 SUPER 8G), which resulted in NaN for all reported values.

Figure 4 shows that BERT-RBP achieved the highest mean score (0.882), followed by iDeepS (0.848), GraphProt (0.846), RNAelem (0.726), RNApromo (0.621), and CMfinder (0.585). Among CFG-based tools, RNAelem performed best, but was outperformed by DNN and SVM models. This discrepancy may be attributed to biological sequence data embodying unmodeled features such as 3D structures, pseudoknots, and complex sequence patterns over long regions, which are better handled by models with extensive parameters.

Furthermore, the prediction accuracy for RNAelem was calculated only for top-ranked motifs, although RNAelem learns 135 search patterns during the optimization process. Employing these models as weak learners could enhance the accuracy of binding predictions. In this study, we trained a model dedicated to binding prediction using 135 suboptimal motifs as 135-dimensional explanatory variables with CatBoost, a gradient boosting method, and showed improvement in AUROC (0.726→0.867) (Figure). Although still underperforming compared to BERT-RBP (0.882), the results indicates that our discovered motifs have significant contribution to RBP-RNA interaction. This experiment is detailed in the Supplementary “Ensemble learning using suboptimal motifs” section.

### 3.3 Comparison of motif visualizations

**Figure 6.**
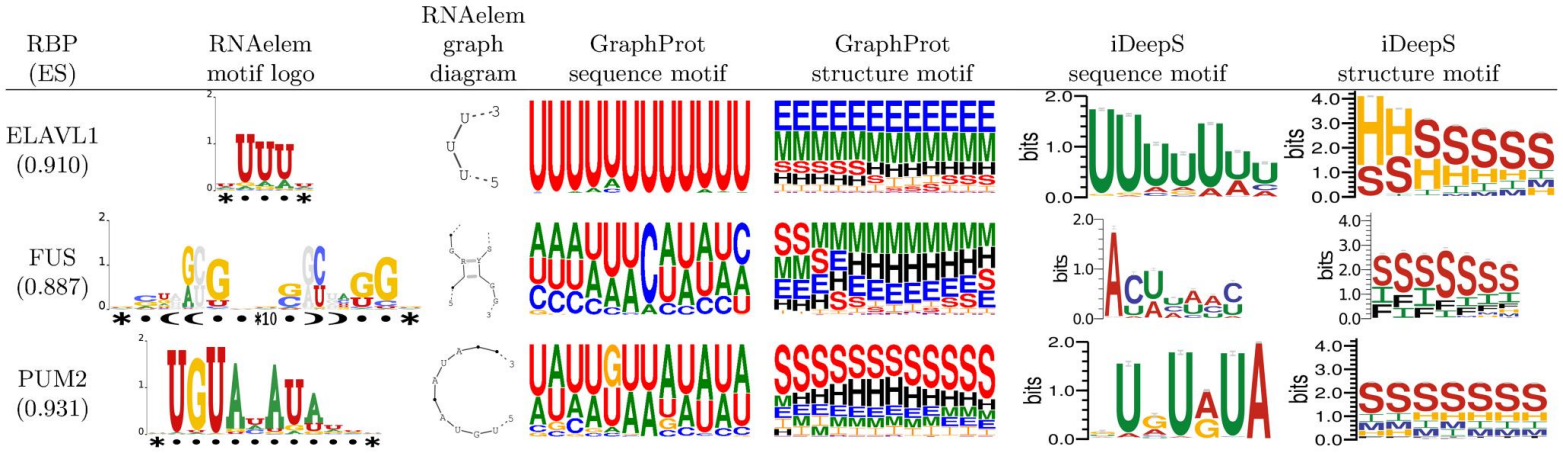
Comparison of motif visualizations among motif search tools. The figure displays motif logos for common RBPs generated by various sequence-structure motif discovery tools. For GraphProt (Maticzka et al., 2014) and iDeepS (Pan et al., 2018), the motif logos have been retrieved from their original publications.

Beyond predicting binding regions, we delve into the primary focus of this study: Motif discovery around these regions. We compared the motif visualizations from our analysis with those previously reported (Fig. 6). This comparison included GraphProt (Maticzka *et al.* 2014) and iDeepS (Pan *et al.* 2018), two tools that provide sequence-structure motif visualization and have conducted RBP-binding motif analysis in their original publications. For all the nine common RBPs across all studies, refer to Supplementary Fig. S9.

GraphProt and iDeepS generate motifs separately for sequences and structures, whereas RNAelem overlays sequence profiles onto the secondary structure of motifs for clearer correspondence. Furthermore, while GraphProt and iDeepS represent stem regions with a one-dimensional profile denoted by “S”, RNAelem indicates the base-pairing partners that form stem regions with balanced brackets “(” and “)”, along with a base-pair profile. Additionally, RNAelem includes an insertion region, denoted as “*”, allowing for the representation of long-spanning regions that are spatially adjacent due to base-pairing, thus enhancing the motif’s expressive capacity.

### 3.4 Exhaustive motif identification using eCLIP data

Next, we performed de novo motif detection across the entire eCLIP dataset. Unlike in previous comparisons where eCLIP peaks were divided into training and testing datasets, here the entire dataset was used for training. Table 1 shows the mean, indicating a relatively high level of discrimination. For 101 out of 167 RBPs, the best predicted motif included at least one base pair, while for 108 RBPs, it contained an insertion region, highlighting the importance of insertion regions in the motif model. Supplementary Fig. S10 presents the ES for each of the 167 RBPs, grouped based on two criteria: Whether the best motif contains a stem region, and whether it includes an insertion region. The data suggest that motifs with base pairs are likely to include insertion regions, corresponding to high ESs, implying that a binding configuration similar to that depicted in Fig. 1 occurs frequently. Conversely, loop-only motifs are less likely to include insertion regions, suggesting that conserved base-pairing is essential for an RBP to stably bind to separate parts of a target RNA. The motifs for 30 RBPs with the top ESs are depicted in Supplementary Fig. S11.

**Table 1.** Count and mean ES of each motif category.

Motif category	Count	Mean ES
All	167	0.782
With base pair	101	0.797
Without base pair	66	0.759
With insertion region	108	0.790
Without insertion region	59	0.767

The table shows the statistics of predicted motifs for eCLIP database.

### 3.5 Comparison with experimentally validated motifs

To verify the biological validity of the sequence-structure motifs identified by RNAelem, we compared motifs with high ES scores (≥0.794) with experimentally validated ones. The referenced experiments include those that demonstrated loss of RBP binding upon motif deletion, induced binding by motif insertion, and 3D structural resolution via X-ray crystallography or NMR. The results are summarized in Table, showing that the predictions of RNAelem are roughly consistent with the experimentally validated motifs.

Noteworthy examples include specific sequence recognitions by RBPs, such as TARDBP’s recognition of UG repeats (Xiao *et al.* 2011), PUM2’s recognition of UGUANAUA (Lu and Hall 2011), and QKI’s recognition of CUAAC (Teplova *et al.* 2013), which are repeatedly reported due to the ease of analyzing sequence consensus. RNAelem, along with other tools, extracted these motifs with high precision. RNAelem’s outputs were the most comprehensive and accurately reported Shannon information content for each position.

“N-rich sequence” is a term commonly used to denote RBP-targeting preference, including HNRNPC and PTBP1’s U-rich (Oberstrass *et al.* 2005, Cienikova *et al.* 2014), PCBP2’s C-rich (Du *et al.* 2007), HNRNPM’s GU-rich (Datar *et al.* 1993), and ELAVL1 and KHSRP’s AU-rich motifs (Gao *et al.* 1994, Díaz-Moreno *et al.* 2010, Pabis *et al.* 2019). While these motifs reveal underlying base biases, they do not fully account for the specificity of RBP interactions. RNAelem effectively identified consistent motifs and additionally proposed contributions of motif length, repeat units, and secondary structures, thus enhancing the specificity of traditional hypotheses.

For RBPs that prefer secondary structures, such as ELAC2’s interaction with the tRNA acceptor stem (Meynier *et al.* 2024), FUS’s interaction with variable-length stem loops (Loughlin *et al.* 2019, Jutzi *et al.* 2020), TAF15 and HNRNPA1’s recognition of stem loops (Kashyap *et al.* 2015, Morgan *et al.* 2015, Kooshapur *et al.* 2018), and DDX3X’s engagement with double-stranded regions (Song and Ji 2019), RNAelem successfully identified the presence of base pairs in these motifs. Other tools, in contrast, occasionally misclassified these structures as loops. Notably, RNAelem correctly identified the TAF15 motif as a stem region with low sequence specificity and suggested that the FUS motif exhibits base pair specificity within stems.

### 3.6 Stem structure existence may recruit U2AF2

**Figure 7.**
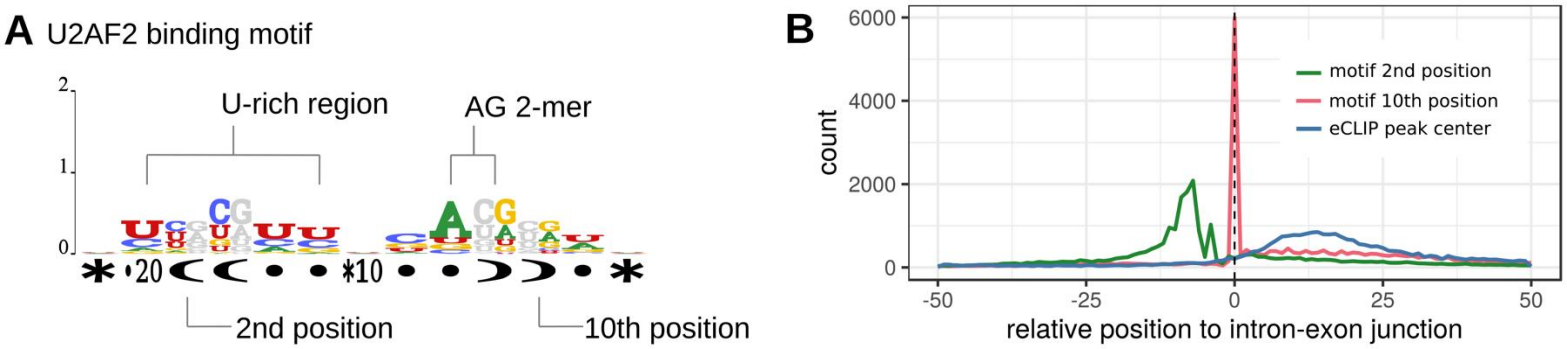
Sequence-structure motifs of U2AF2. (A) U2AF2’s binding motif features a base pair within its structure. (B) Metagene profiles of U2AF2’s binding motif, including eCLIP peaks, around the intron-exon junction.

U2AF2 encodes a 65 kDa subunit of U2AF. Crystallographic analysis revealed that its two domains, RRM1 and RRM2, are connected by a flexible linker, facilitating recognition of variable-length poly-U (Sickmier *et al.* 2006, Mackereth *et al.* 2011). Additionally, the 35 kDa subunit of U2AF recognizes AG dimers near the intron-exon junction (Merendino *et al.* 1999). The intron of the pre-mRNA contains a 15–20 nt U-rich region called the polypyrimidine (Py) tract, which is thought to recruit the spliceosome. Figure 7A suggests that RNAelem has identified the two-domain motif recognized by these subunits. Interestingly, the C or U of the Py tract and the G of the AG dimer appear to form base pairs, suggesting spatial proximity. Gene annotation of the binding regions confirms that the predicted positions of the binding regions and the motifs detected in U2AF2 are enriched near the intron-exon junction (Fig. 7B). eCLIP peaks are predominantly found on the exon side of this junction, whereas the position of the AG dimer aligned with RNAelem was enriched at the junction. These findings indicate that U2AF recognizes both the Py tract and the splicing junction, and their spatial proximity by forming a stem structure is the starting point for splicing. The Py tract is variable in length and poorly conserved, and this has made secondary structure analysis difficult. Thus, this is an excellent example of how base pair profile learning and the introduction of insertion regions produce effective results.

### 3.7 Molecular mechanisms of hnRNP family binding

**Figure 8.**
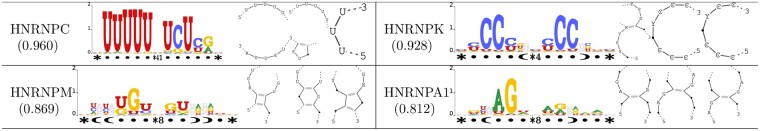
Sequence-structure motifs of HNRNP family. RBP name, ES, optimal motif sequence logo, and top three suboptimal motif graph diagrams are shown.

The hnRNP family is a large family of RBPs that are involved in multiple aspects of nucleic acid metabolism, including alternative splicing, mRNA stabilization, transcriptional regulation, and translational regulation (Geuens *et al.* 2016). HNRNPCs are known to bind to U-rich target molecules using the RRM domain (Cienikova *et al.* 2014). This target region is enriched in pre-messenger, alternatively spliced exons. RNAelem reported the sequence motif UCUYR downstream of the U-rich region, with a gap of approximately 40 bases between them (Fig. 8). This 5-mer is consistent with the sequence profile that is conserved downstream of UUUGAGACAG, one of the consensus sequences of the 3’ acceptor of the alternative splicing site (Qu *et al.* 2017). Indeed, the UUUGAGACAG sequence appeared in 138 of the 1000 binding sites selected as positive examples. *Qu et al.* showed a match between these consensus sequences and the targets of miRNAs mediating alternative splicing (Qu *et al.* 2017). This is supported by the fact that RNAelem reported that the UCUYR region is single-stranded and accessible to miRNAs.

The multifunctional protein HNRNPA1 binds specifically to stem-loops (Damgaard *et al.* 2002). Co-crystallographic analysis of the UP1 domain with RNA showed that AG dimer are specifically recognized (Morgan *et al.* 2015). RNAelem identified these two bases at the 3’ boundary of the stem-loop in all three top motif candidates (Fig. 8). Given the brevity of the recognition sequences, their binding specificity may be better explained by the secondary structures involved. To the best of our knowledge, this is the first report of the HNRNPA1 binding motif being inferred with such high resolution.

## 4 Conclusion

We have developed RNAelem, a tool for detecting sequence-structure motifs from unaligned RNA sequence sets with greater accuracy than previously possible. This tool is particularly effective in identifying secondary structural motifs in the binding regions of RBPs. Benchmarking with simulated data demonstrated that RNAelem accurately learns both conserved sequences and local structures of motifs. When applied to CLIP-Seq data, it supported or enhanced motif models for various RBPs. For instance, it suggested that the targeting of the splicing factor U2AF2 depends on the stem structures with variable lengths present at splicing junctions. The motifs reported by RNAelem are highly interpretable and will be a valuable analytical tool for elucidating post-transcriptional regulation.

## Supplementary Material

vbae144_Supplementary_Data

## Data Availability

A C++ implementation of RNAelem (https://github.com/iyak/RNAelem) and a dedicated sequence logo generator (https://github.com/iyak/RNAlogo) are available under the MIT license on GitHub. The simulated datasets used in the experiments and the motifs detected for all RBPs included in eCLIP are available from https://github.com/iyak/RNAelem-data. The eCLIP datasets analyzed were obtained from the ENCODE project database (https://www.encodeproject.org) (Van Nostrand et al., 2020). See the main text for the processing methods.
