# Supplementary material for: RNAelem: an algorithm for discovering sequence-structure motifs in RNA bound by RNA-binding proteins
Source: Bioinform Adv. 2024 Sep 28;4(1):vbae144. doi: 10.1093/bioadv/vbae144 (PMC11471262; doi:10.1093/bioadv/vbae144)
Supplement: vbae144_Supplementary_Data [file vbae144_supplementary_data.zip › Supplementary Materials.pdf]

Supplementary materials for "RNAelem: An Algorithm for Discovering Sequence-Structure Motifs in RNA Bound by RNA-Binding Proteins"

1 Methods

1.1 Energy model for RNA secondary structure

The dynamics of RNA folding can be modeled using a CFG that emits bases and base pairs. We adopted the Boltzmann energy distribution that is defined by the Turner energy model (Mathews et al., 1999). Of the CFGs that are compatible with their energy parameters, we followed the Rfold model, which sets an upper limit on the base pair distance, or maximal span W, to reduce computational complexity (Kiryu et al., 2008). This constraint is convenient for the modeling of local structural motifs.

The Rfold model employs an unambiguous grammar; in it, one secondary structure corresponds to one parse tree. The free energy of a secondary structure is obtained by summing the free energy changes that are associated with the state transitions of the corresponding parse tree. To see this, let σ be a parse tree that is associated with a secondary structure of sequence x. The probability of the parse tree σ given a sequence x is proportional to the following function,

$$\begin{aligned}\tilde{P}_{\texttt{energy}}(\sigma \mid x) &= \prod_{p\in\sigma} \tilde{P}_{\texttt{energy}}(p \mid x) \\ &= \prod_{p\in\sigma} \exp\left(-\frac{1}{kT}\Delta G(p,x)\right)\end{aligned} \tag{S1}$$

where k is Boltzmann's constant, T is temperature and ΔG is the free energy change associated with state transition p.

In the following, we will briefly review the grammar of the Rfold model. Let $x = \{\texttt{A},\texttt{C},\texttt{G},\texttt{U}\}^L$ be an RNA sequence of length L. The non-terminal symbols V_1, the terminal symbols T_1, the state

transition rules P_1, and the start symbol S_1 of the CFG are given as follows.

$$
\begin{aligned}
\text{Rfold} &= (V_1, T_1, P_1, S_1) \\
V_1 &= \{\texttt{Outer}, \texttt{Stem}, \texttt{StemEnd}, \texttt{Loop}, \texttt{Multi}, \texttt{MultiBif}, \texttt{Multi1}, \texttt{Multi2}\} \\
T_1 &= \{\texttt{A}, \texttt{C}, \texttt{G}, \texttt{U}\} \\
P_1 &= \left\{\begin{array}{ll}
\texttt{Outer} & \rightarrow \epsilon \mid \texttt{Outer} \cdot a \mid \texttt{Outer} \cdot \texttt{Stem}, \\
\texttt{Stem} & \rightarrow b_1 \cdot \texttt{Stem} \cdot b_2 \mid b_1 \cdot \texttt{StemEnd} \cdot b_2, \\
\texttt{StemEnd} & \rightarrow \texttt{Loop} \mid \texttt{Loop} \cdot \texttt{Stem} \cdot \texttt{Loop} \mid \texttt{Multi}, \\
\texttt{Loop} & \rightarrow \epsilon \mid \texttt{Loop} \cdot a \\
\texttt{Multi} & \rightarrow a \cdot \texttt{Multi} \mid \texttt{MultiBif}, \\
\texttt{MultiBif} & \rightarrow \texttt{Multi1} \cdot \texttt{Multi2}, \\
\texttt{Multi1} & \rightarrow \texttt{MultiBif} \mid \texttt{Multi2}, \\
\texttt{Multi2} & \rightarrow \texttt{Multi2} \cdot a \mid \texttt{Stem}
\end{array}\right\} \\
& a \in \texttt{A}, \texttt{C}, \texttt{G}, \texttt{U} \\
& (b_1, b_2) \in (\texttt{C}, \texttt{G}), (\texttt{G}, \texttt{C}), (\texttt{A}, \texttt{U}), (\texttt{U}, \texttt{A}), (\texttt{G}, \texttt{U}), (\texttt{U}, \texttt{G}) \\
S_1 &= \texttt{Outer}
\end{aligned} \tag{S2}
$$

Each secondary structure of sequence x corresponds to one Rfold's parse tree. For example, in the toy example sequence `CCAAAG`, where the possible secondary structures are "`......`", "`(....)`", "`.(...)`", the following state transition set represents the secondary structure "`.(...)`"

$$
\sigma = \left\{\begin{array}{ll}
\langle \texttt{Outer}, 0, 6 \rangle & \rightarrow \langle \texttt{Outer}, 0, 1 \rangle \langle \texttt{Stem}, 1, 6 \rangle, \\
\langle \texttt{Outer}, 0, 1 \rangle & \rightarrow \langle \texttt{Outer}, 0, 0 \rangle \, \texttt{C}, \\
\langle \texttt{Outer}, 0, 0 \rangle & \rightarrow \epsilon, \\
\langle \texttt{Stem}, 1, 6 \rangle & \rightarrow \texttt{C} \, \langle \texttt{StemEnd}, 2, 5 \rangle \, \texttt{G}, \\
\langle \texttt{StemEnd}, 2, 5 \rangle & \rightarrow \langle \texttt{Loop}, 2, 5 \rangle, \\
\langle \texttt{Loop}, 2, 5 \rangle & \rightarrow \langle \texttt{Loop}, 2, 4 \rangle \, \texttt{A}, \\
\langle \texttt{Loop}, 2, 4 \rangle & \rightarrow \langle \texttt{Loop}, 2, 3 \rangle \, \texttt{A}, \\
\langle \texttt{Loop}, 2, 3 \rangle & \rightarrow \langle \texttt{Loop}, 2, 2 \rangle \, \texttt{A}, \\
\langle \texttt{Loop}, 2, 2 \rangle & \rightarrow \epsilon
\end{array}\right\} \tag{S3}
$$

where tuple $\langle v, i-1, j \rangle$ represents that sub-sequence $x_i \cdots x_j$ is emitted downstream of nonterminal symbol $v \in V_1$. Similarly, there are unique parse trees σ for the other two secondary structures.

Each state transition $p \in \sigma$ is associated with a potential $\tilde{P}_{\texttt{energy}}(p \mid x)$ defined by the Turner energy model (Mathews et al., 1999). Then the Boltzmann distribution of a parse tree σ is given by,

$$
P_{\texttt{energy}}(\sigma \mid x) = \frac{\tilde{P}_{\texttt{energy}}(\sigma \mid x)}{Z_{\texttt{energy}}(x)} \tag{S4}
$$

$$
Z_{\texttt{energy}}(x) = \sum_{\sigma} \tilde{P}_{\texttt{energy}}(\sigma \mid x) \tag{S5}
$$

where $Z_{\texttt{energy}}(x)$ represents the partition function.

1.2 Profile CFG model

A Profile CFG is constructed for each search pattern $\mu = \{\texttt{.}, \texttt{(}, \texttt{)}, \texttt{*}\}^M$ where M is the length of the search pattern. This approach is inspired by previous studies that have used CFGs to study RNA base pairing profiles (Eddy, 2002; Fang et al., 2008). Figure S1 presents the definition of the Profile CFG, using search pattern "`*(.(*))*`" as an example. Hidden states of the Profile CFG are labeled with the intervals $[m, n]$, where m and n represent position indices of the pattern μ

$(0 \leq m \leq n \leq M)$. Only intervals that contain a valid substructure in $\mu_m \cdots \mu_{n-1}$ are assigned a hidden state. The hidden states transition to each other as they emit a base to the left or the right or emit a base pair to both sides. For each dot and bracket of pattern μ we associate an independent base and base-pair profile parameters θ. If $m = n$, the state is extinguished by emitting null base ϵ.

Inspired by the concept of "abstract shapes" introduced by Steffen et al., in which single-stranded regions and stem regions of different lengths are consolidated into a single symbol for efficient classification of RNA structures (Steffen et al., 2006), we allow for self-transitions for each hidden state corresponding to a dot or bracket in order to account for occasional fluctuation of stem and loop lengths. We assign a penalty $e^{\omega} = 0.1$ to each self-transition of hidden states to limit the frequency of self-transitions.

The emissions by Profile CFG can be categorized into four types: (1) single-base emission from loop regions within motifs, (2) single-base emission from insertion regions, (3) base-pair emission from stem regions within motifs, and (4) two-base emission from insertion regions. The index sets for profile θ used in each scenario, namely H_{loop}, H^*_{loop}, H_{pair}, and H^*_{pair}, are defined as:

$$\begin{aligned}
H_{\text{loop}} &= \{i \mid \mu_i = .\} \\
H^*_{\text{loop}} &= \{i \mid \mu_i = *\} \\
H_{\text{pair}} &= \{i, j \mid i < j, \mu_i = (, \mu_j =), D_{i+1} = D_j\} \\
H^*_{\text{pair}} &= \{i, j \mid i \leq j, \mu_i = *, \mu_j = *, D_{i+1} = D_j\}
\end{aligned} \tag{S6}$$

where D_i denotes the "scope index," an identifier for the closest base pair enclosing the region within the search pattern μ, as determined by Algorithm S1.

Now we define the matching score of the Profile CFG. Profile CFG is a generative model that outputs RNA sequences with state transitions, while also being a parser that can describe the process of outputting a given RNA sequence. As state transitions have a tree structure, their instances are called parse trees. The parse tree ϕ is represented by a set of state transitions, that are scored by a base and a base pair profile θ. The probability of ϕ given a sequence x is proportional to

$$\begin{aligned}
\tilde{P}_{\texttt{profile}}(\phi \mid \mu, \theta, x) = \exp\Big(&\sum\nolimits_{\substack{i \in H_{\text{loop}}, \\ a \in B_1}} \theta_{i,a} N_{i,a}(\phi, x) \\
&+ \sum\nolimits_{\substack{(i,j) \in H_{\text{pair}}, \\ (b_1, b_2) \in B_2}} \theta_{i,j,b_1,b_2} N_{i,j,b_1,b_2}(\phi, x) \\
&+ \sum\nolimits_{a \in B_1} \theta_{0,a} N_{0,a}(\phi, x) \\
&+ \omega N(\phi, x) \Big)
\end{aligned} \tag{S7}$$

where $B_1 = \{\texttt{A}, \texttt{C}, \texttt{G}, \texttt{U}\}$, and $B_2 = \{\texttt{AU}, \texttt{CG}, \texttt{GC}, \texttt{GU}, \texttt{UA}, \texttt{UG}\}$. Here, $N(\phi, x)$ represents the total number of self-transitions in ϕ. $N_{i,a}(\phi, x)$ and $N_{i,j,b_1,b_2}(\phi, x)$ count the emissions of base a by loop symbol μ_i, and base pairs b_1, b_2 by μ_i, μ_j, respectively. $N_{0,a}(\phi, x)$ accounts for the base emissions by insertion regions and also includes the base pair emissions, treating the bases at both ends individually:

$$\begin{aligned}
N_{0,a}(\phi, x) = &\sum\nolimits_{i \in H^*_{\text{loop}}} N_{i,a}(\phi, x) \\
&+ \sum\nolimits_{\substack{(i,j) \in H^*_{\text{pair}}, \\ b \in B_1}} N_{i,j,a,b}(\phi, x) + N_{i,j,b,a}(\phi, x)
\end{aligned} \tag{S8}$$

Moving forward, the grammar of the Profile CFG model will be formalized. Given a search

Supplementary Algorithm S1 Calculation of scope index D for a given search pattern μ

$D_0 = 0$
$stack \leftarrow \{0\}$
$maxIndex \leftarrow 0$
for $i \leftarrow 1, \dots, |\mu|$ **do**
  if $\mu_{i-1} = \texttt{(}$ **then**
    $stack.push(maxIndex + 1)$
    $maxIndex \leftarrow maxIndex + 1$
  else if $\mu_{i-1} = \texttt{)}$ **then**
    $stack.pop()$
  end if
  $D_i \leftarrow stack.back()$
end for

Supplementary Algorithm S2 Compute left emit set E_l and right emit set E_r that represent the set of transitions between the positions in search pattern μ

$E_l \leftarrow \{\}$
$E_r \leftarrow \{\}$
for $i \leftarrow 1, \dots, |\mu|$ **do**
  if $\mu_{i-1} = \texttt{.}$ **then**
    $E_l \leftarrow E_l \cup \{(i-1, i)\}$
    $E_r \leftarrow E_r \cup \{(i, i-1)\}$
  else if $\mu_{i-1} = \texttt{(}$ **then**
    $E_l \leftarrow E_l \cup \{(i-1, i)\}$
  else if $\mu_{i-1} = \texttt{)}$ **then**
    $E_r \leftarrow E_r \cup \{(i, i-1)\}$
  else
    $E_l \leftarrow E_l \cup \{(i-1, i-1), (i-1, i), (i-1, i+1)\}$
    $E_r \leftarrow E_r \cup \{(i, i), (i, i-1), (i, i-2)\}$
  end if
end for

pattern $\mu = \mu_1 \cdots \mu_M$, its associated Profile CFG is defined as follows,

$$
\begin{aligned}
\text{Profile CFG} &= (V_2, T_2, P_2, S_2) \qquad \text{(S9)}\\
V_2 &= \{[m,n] \mid m \leq n, D_m = D_n\}\\
T_2 &= \{\texttt{A}, \texttt{C}, \texttt{G}, \texttt{U}\}\\
P_2 &= \left\{ [m,n] \;\rightarrow\; \begin{array}{ll}
[m,v]\cdot a \,| & \text{if } D_m = D_v = D_n \;\&\; (n,v) \in E_r\\
a \cdot [u,n] \,| & \text{if } D_m = D_u = D_n \;\&\; (m,u) \in E_l\\
b_1 \cdot [u,v] \cdot b_2 \,| & \text{if } (m,u) \in E_l \;\&\; (n,v) \in E_r\\
 & \;\& \; (\mu_{u-1}, \mu_v) \in \{(\texttt{(},\texttt{)}), (\texttt{*},\texttt{*})\}\\
[m,n] \,| & \\
[m,u][u,n] \,| & \text{if } D_m = D_u = D_n\\
[m,u][u,v][v,n] \,| & \text{if } D_m = D_u = D_v = D_n\\
\epsilon & \text{if } m = n
\end{array} \right\}\\
& \quad a \in \texttt{A}, \texttt{C}, \texttt{G}, \texttt{U}\\
& \quad (b_1, b_2) \in (\texttt{C},\texttt{G}), (\texttt{G},\texttt{C}), (\texttt{A},\texttt{U}), (\texttt{U},\texttt{A}), (\texttt{G},\texttt{U}), (\texttt{U},\texttt{G})\\
S_2 &= [0, |\mu|]
\end{aligned}
$$

where array $D = (D_0, D_1, \dots, D_M)$ represents "scope index" for each interval boundary in a search pattern. A scope index is a unique identifier assigned to both the global scope and local scopes delineated by paired "`(`" and "`)`". In other words, these are uniformly allocated to positions enclosed

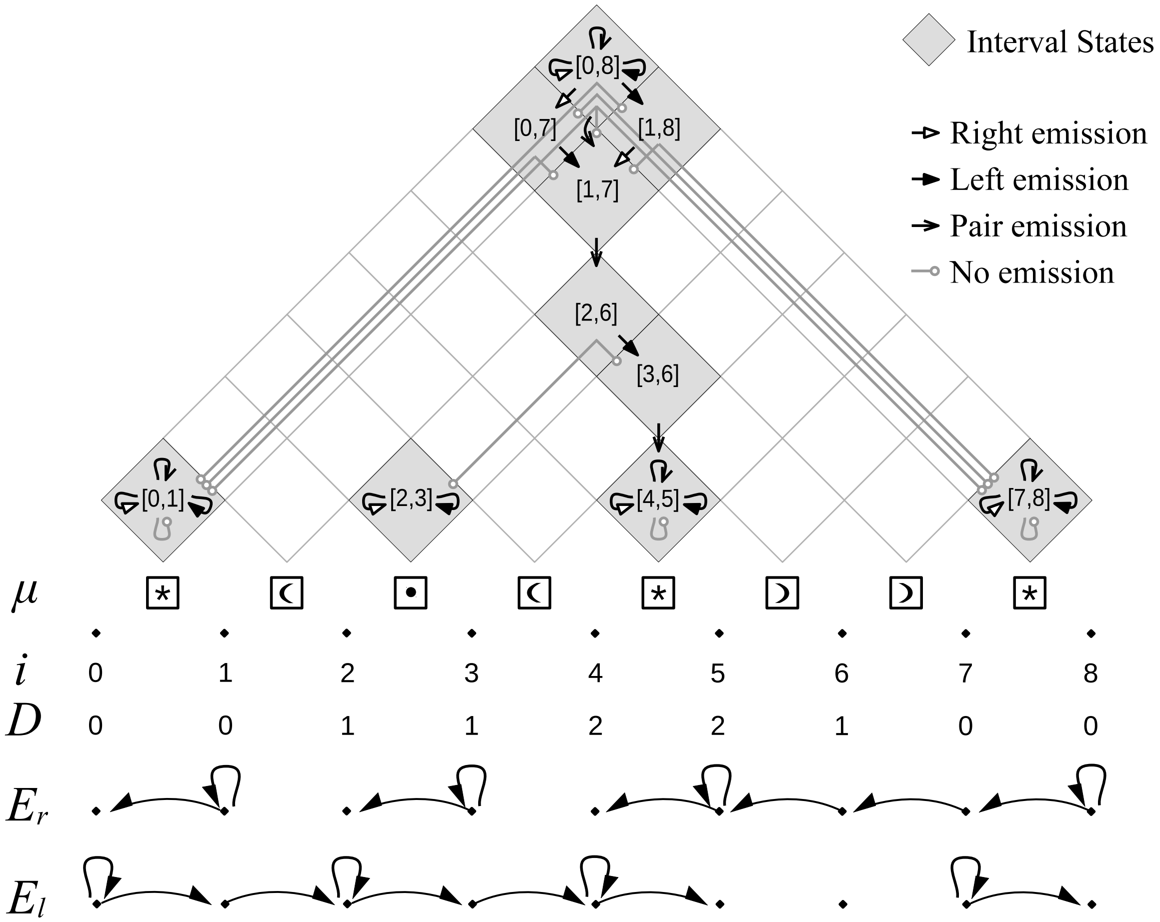

Supplementary Figure S1: **Illustrative example of Profile CFG corresponding to the search pattern** $\mu =$ "`*(.(*))*`" Scope index D and emit sets E_r, E_l are determined by algorithms S1 and S2 for the given search pattern μ. Interval states are defined for intervals $[i,j]$ where $D_i = D_j$. Transitions between these states are then specified according to conditions outlined in Equation S9.

by an identical pair of brackets and have the same nesting levels. The stack-based algorithm used to compute these scope index is detailed in Algorithm S1.

The elements in V_2 are called interval states. E_l and E_r, the left emit set and right emit set, form the transition sets between the positions in search pattern μ. They determine if the transition between interval states can emit bases to left or right. Left emit set E_l and right emit set E_r are computed by Algorithm S2.

For example, the Profile CFG corresponding to the search pattern "`*(.(*))*`", which is also the example used in the main article, is defined as follows (see also Figure S1).

$$
\begin{aligned}
\mu &= \texttt{*(.(*))*} && \text{(S10)}\\
\text{Profile CFG} &= (V_2, T_2, P_2, S_2)\\
V_2 &= \{[0,1],[0,7],[0,8],[1,7],[1,8],[2,3],[2,6],[3,6],[4,5],[7,8]\}\\
T_2 &= \{\texttt{A},\texttt{C},\texttt{G},\texttt{U}\}\\
P_2 &= \left\{\begin{array}{ll}
[0,8] & \to a\cdot[0,8] \mid [0,8]\cdot a \mid b_1\cdot[0,8]\cdot b_2 \mid a\cdot[1,8] \mid [0,7]\cdot a \mid\\
 & \quad [0,8] \mid [0,1][1,8] \mid [0,7][7,8] \mid [0,1][1,7][7,8]\\
[0,7] & \to a\cdot[1,7] \mid [0,7]\cdot a \mid [0,1][1,7]\\
[1,8] & \to [1,7]\cdot a \mid [1,7][7,8]\\
[0,1] & \to \epsilon \mid a\cdot[0,1] \mid [0,1]\cdot a \mid b_1\cdot[0,1]\cdot b_2 \mid\\
 & \quad [0,1] \mid [0,1][0,1] \mid [0,1][0,1][0,1]\\
[7,8] & \to \epsilon \mid a\cdot[7,8] \mid [7,8]\cdot a \mid b_1\cdot[7,8]\cdot b_2 \mid\\
 & \quad [7,8] \mid [7,8][7,8] \mid [7,8][7,8][7,8]\\
[1,7] & \to b_1\cdot[2,6]\cdot b_2\\
[2,6] & \to a\cdot[3,6] \mid [2,3][3,6]\\
[2,3] & \to \epsilon \mid a\cdot[2,3] \mid [2,3]\cdot a\\
[3,6] & \to b_1\cdot[4,5]\cdot b_2\\
[4,5] & \to \epsilon \mid a\cdot[4,5] \mid [4,5]\cdot a \mid b_1\cdot[4,5]\cdot b_2 \mid\\
 & \quad [4,5] \mid [4,5][4,5] \mid [4,5][4,5][4,5]
\end{array}\right\}\\
& a \in \texttt{A},\texttt{C},\texttt{G},\texttt{U}\\
& (b_1,b_2) \in (\texttt{C},\texttt{G}),(\texttt{G},\texttt{C}),(\texttt{A},\texttt{U}),(\texttt{U},\texttt{A}),(\texttt{G},\texttt{U}),(\texttt{U},\texttt{G})\\
S_2 &= [0,8]
\end{aligned}
$$

Let ϕ be a parse tree for the Profile CFG. We associate each state transition $p \in \phi$ with a potential function. If a transition emits a base pair or an unpaired base, then an associated potential $\exp(\theta)$ is multiplied. We allocate an independent base profile parameter $\theta_{i,\texttt{A}}, \theta_{i,\texttt{C}}, \theta_{i,\texttt{G}}, \theta_{i,\texttt{U}}$ for each unpaired motif base with $\mu_i =$ "`.`". Likewise, we allocate an independent base pair profile $\theta_{i,j,\texttt{A},\texttt{U}}, \theta_{i,j,\texttt{U},\texttt{A}}, \theta_{i,j,\texttt{C},\texttt{G}}, \theta_{i,j,\texttt{G},\texttt{C}}, \theta_{i,j,\texttt{G},\texttt{U}}, \theta_{i,j,\texttt{U},\texttt{G}}$ for each motif base pair μ_i and μ_j. On the other hand, all of the insertion regions share a single background base profile parameter.

In state transitions P_2, we included self-loops for "`(`", "`)`", "`.`". This allows each node to match different loop and stem lengths. For example, search pattern "`*(.)*`" now matches any hairpin loop. This allows a single motif to account for fluctuations in loop and stem lengths in different binding regions. It also enables the pattern size to be compressed when a motif contains contiguous unpaired bases with similar base profiles. However, this technique may distort the probability distribution of the model, as it increases the number of different matching patterns for the same motif location. For a compromise, a potential $\exp(\omega)$ (default 0.1) is given to each self-loop transition to penalize the inclusion of many self-loop transitions.

1.2.1 Comparison with Infernal's Profile CFG

Profile CFGs, utilized by Infernal, facilitate the extraction of sequence-structure conservation from RNA sequence alignments (Nawrocki and Eddy, 2013). Infernal employs covariance models to describe RNA secondary structures (Eddy, 2002).

Infernal primarily classifies and searches structured RNA gene families, constructing RNA secondary structure models using evolutionary covariation of base-pairs from multiple RNA sequence alignments. Conversely, the RBP binding motifs targeted are smaller than those assumed by Infernal and typically lack evolutionary conservation among different binding sites, complicating the use of multiple alignments as input.

RNAelem, therefore, employs an energy model of secondary structure to construct models from *u*naligned sequence sets, rather than relying on multiple alignments. This approach results in higher computational costs; while Infernal deterministically and quickly determines secondary structure and parameters using base-pair covariations, RNAelem optimizes parameters for all search patterns

using gradient descent to select the optimal pattern. Furthermore, for determining motif positions in sequences, Infernal uses dynamic programming to align the CFG of the structural motif with the primary sequence, whereas RNAelem aligns the CFG of the structural motif with the energy model CFG on the sequence, further increasing computational demands.

One important difference is that our profile CFG does not allow for state deletions. One reason for this is that our search patterns are about 10 bases long, and allowing deletions would significantly reduce the specificity of sequence matching by losing self-identity. More seriously, allowing deletions would make search patterns to match entirely different secondary structure topologies. For example, if one base of a base pair within a stem is deleted, a bulge loop is formed. Conversely, if a loop base of a bulge loop is deleted, two stems merge into one long stem. In more extreme cases, deletions of CFG states could lead to the creation, disappearance, and merging of loops and stems, and they result in a single search pattern to match entirely different secondary structure topologies from when there are no deletions (whereas repeating a state through self-transitions do not change the topology or RNA shape of the secondary structure being matched). Our algorithm sums up the Boltzmann factor of every secondary structure that matches a search pattern, but allowing deletions would sum up the Boltzmann factors of entirely different secondary structure topologies and energy structures, making the score function unclear. Additionally, in visualizing motifs, showing both a base-pair profile and a bulge loop profile at each motif base-pair position would significantly reduce interpretability. Therefore, if deletions are allowed, a very large penalty must be applied to the transition probability, which would reduce its effect on the results. Given these considerations, we concluded that the benefits of incorporating deletions are very small and decided not to include them in the model.

1.3 Integration of profile and energy model

RNAelem is a large CFG that combines the Profile CFG and Rfold. Its states consist of a pair of states from the two CFGs. Let $[m, n]$ and s denote a state of Profile CFG and Rfold. Then the transition between the coupled states is denoted by $([m, n], s) \to ([m', n'], s')$. We only consider the transitions that its decomposed state transitions $s \to s'$ and $[m, n] \to [m', n']$ have the same base and base pair emission. Following this constraint, the coupled CFG acts as a parser of the RNA sequence, and its parse tree contains both the parse tree σ from the Rfold and the parse tree ϕ from the Profile CFG. We denote RNAelem's parse tree $\sigma \otimes \phi$, or simply τ. The parse tree is scored using both base and base pair profile parameters, as well as the Turner energy parameters. The Boltzmann probability of this coupled parse tree $\sigma \otimes \phi$ is proportional to

$$\tilde{P}_{\texttt{couple}}(\sigma \otimes \phi \mid \mu, \theta, \lambda, x) = \tilde{P}_{\texttt{energy}}(\sigma \mid x)^{\lambda} \tilde{P}_{\texttt{profile}}(\phi \mid \mu, \theta, x), \tag{S11}$$

where λ is a parameter that balances the relative strengths of the motif match and of secondary structure stability, which is fitted to the data during the training. To treat parsing by the coupled CFG as a probabilistic model, we employed a conditional random field (CRF). Thus, the probability of a parse tree $\sigma \otimes \phi$ can be represented as follows:

$$P_{\texttt{couple}}(\tau \mid \nu, x) = \frac{\tilde{P}_{\texttt{couple}}(\tau \mid \nu, x)}{Z(\nu, x)} \tag{S12}$$

$$Z(\nu, x) = \sum_{\tau \in \Phi(x)} \tilde{P}_{\texttt{couple}}(\tau \mid \nu, x) \tag{S13}$$

where Z and $\Phi(x)$ are the partition function and all of the possible parse trees of sequence x, respectively.

We now formalize the CFG grammar of RNAelem as the direct product of the Rfold CFG and

the Profile CFG.

$$
\begin{aligned}
\text{RNAelem} &= (V, T, P, S) \\
V &= V_1 \times V_2 \\
&= \{(s,t); s \in V_1, t \in V_2\} \\
T &= \{\texttt{A}, \texttt{C}, \texttt{G}, \texttt{U}\} \\
P &= \left\{ \begin{array}{lll} & a(u,v)| & \text{if } s \to a \cdot u \in P_1 \ \& \ t \to a \cdot v \in P_2 \\ & (u,v)a| & \text{if } s \to u \cdot a \in P_1 \ \& \ t \to v \cdot a \in P_2 \\ & b_1(u,v)b_2| & \text{if } s \to b_1 \cdot u \cdot b_2 \in P_1 \ \& \ t \to b_1 \cdot v \cdot b_2 \in P_2 \\ \text{(s,t)} & \to (u,v)| & \text{if } s \to u \in P_1 \ \& \ t \to v \in P_2 \\ & (u_1,v_1)(u_2,v_2)| & \text{if } s \to u_1 u_2 \in P_1 \ \& \ t \to v_1 v_2 \in P_2 \\ & (u_1,v_1)(u_2,v_2)(u_3,v_3)| & \text{if } s \to u_1 u_2 u_3 \in P_1 \ \& \ t \to v_1 v_2 v_3 \in P_2 \\ & \epsilon & \text{if } s \to \epsilon \in P_1 \ \& \ t \to \epsilon \in P_2 \end{array} \right\} \\
& a \in \texttt{A}, \texttt{C}, \texttt{G}, \texttt{U} \\
& (b_1, b_2) \in (\texttt{C},\texttt{G}), (\texttt{G},\texttt{C}), (\texttt{A},\texttt{U}), (\texttt{U},\texttt{A}), (\texttt{G},\texttt{U}), (\texttt{U},\texttt{G}) \\
S &= (S_1, S_2)
\end{aligned} \tag{S14}
$$

Since each state transition rule in P can be decomposed into Rfold's and Profile CFG's, a parse tree of RNAelem can also be decomposed into Rfold's parse tree σ and Profile CFG's parse tree ϕ. For each transition, we associate the product of the potentials of the two CFGs. Then the potential of parse tree $\sigma \otimes \phi$ is given by,

$$
\begin{aligned}
\tilde{p}_{\texttt{couple}}(\tau \mid \nu, x) &= \tilde{p}_{\texttt{energy}}(\sigma \mid x)^{\lambda} \tilde{p}_{\texttt{profile}}(\phi \mid \mu, \theta, x) \\
&= \prod_{p \in \sigma, q \in \phi} \tilde{p}_{\texttt{energy}}(p \mid x)^{\lambda} \tilde{p}_{\texttt{profile}}(q \mid \mu, \theta, x) \\
&= \exp\big(\lambda \gamma(\sigma \mid x) + \textstyle\sum_{\substack{i \in h_{\texttt{loop}}, \\ a \in b_1}} \theta_{i,a} n_{i,a}(\phi, x) \\
&\quad + \textstyle\sum_{\substack{i \in h^*_{\texttt{loop}}, \\ a \in b_1}} \theta_{0,a} n_{i,a}(\phi, x) \\
&\quad + \textstyle\sum_{\substack{(i,j) \in h_{\texttt{pair}}, \\ (b_1,b_2) \in b_2}} \theta_{i,j,b_1,b_2} n_{i,j,b_1,b_2}(\phi, x) \\
&\quad + \textstyle\sum_{\substack{(i,j) \in h^*_{\texttt{pair}}, \\ (b_1,b_2) \in b_2}} (\theta_{0,b_1} + \theta_{0,b_2})\, n_{i,j,b_1,b_2}(\phi, x) \\
&\quad + \omega n(\phi, x)\big)
\end{aligned} \tag{S15}
$$

where $\gamma(\sigma \mid x) = -\Delta G(\sigma, x)/kT$. We scaled the potential of the secondary structure σ by λ to balance the potentials of two CFGs. Further, λ is optimized in the training phase together with profile parameters θ.

Lastly, we will introduce the advanced features of the balance factor λ. As shown in Equation S1 and S11, the potential of parse tree τ is given by,

$$
\tilde{P}_{\texttt{couple}}(\tau \mid \nu, x) = \tilde{P}_{\texttt{energy}}(\sigma \mid x)^{\lambda} \tilde{P}_{\texttt{profile}}(\phi \mid \mu, \theta, x) \tag{S16}
$$

$$
\tilde{P}_{\texttt{energy}}(\sigma \mid x) = \exp\left\{-\frac{1}{kT} \Delta G(\sigma, x)\right\} \ \text{[kcal/mol]} \tag{S17}
$$

where λ scales potential $\tilde{P}_{\texttt{energy}}(\sigma \mid x)$ to balance it with $\tilde{P}_{\texttt{profile}}(\phi \mid \theta, x)$. We expect that the secondary structure of the motif regions is often more stable than the stability outside of the motif region. Hence, to emphasize the potential of the conserved secondary structure in the motif region and to learn them separately, we split λ into λ_{mt} for the inside of the motif and λ_{bg} for the outside

of the motif.

$$
\begin{aligned}
\lambda^{\dagger}(\tau) &= \frac{\sum_{p\in\tau_{\mathrm{mt}}} \Delta G(p,x)}{\Delta G(\tau,x)}\lambda_{\mathrm{mt}} + \frac{\sum_{p\in\tau_{\mathrm{bg}}} \Delta G(p,x)}{\Delta G(\tau,x)}\lambda_{\mathrm{bg}} \qquad \text{(S18)}\\
\tau_{\mathrm{mt}} &= \{\langle (s,[m,n]), i, j\rangle \to \text{any} \mid \mu_m, \mu_{n-1} \neq *, *, *\}\\
\tau_{\mathrm{bg}} &= \tau \setminus \tau_{mt}
\end{aligned}
$$

where τ_{mt} represents the set of transitions inside the motif, and τ_{bg} for those outside the motif. If a state transition emits background base(s), parameter λ_{bg} is used. Otherwise, parameter λ_{mt} is used. Since λ appears in the potential in the form of ratio λ/kT, the local λ can be interpreted as providing different temperatures in the motif region and the background region. At high temperatures, RNA undergoes a transition from one secondary structure to another, less dependent of free energy. While at low temperatures, secondary structures with lower free energies tend to stay unchanged. Hence ratio $\lambda_{\mathrm{bg}}/\lambda_{\mathrm{mt}}$ represents the stability of the motif structure relative to the background region.

1.4 Parameter fitting method

In the learning step, the base and base-pair profile θ and balancer λ are learned to maximize the log likelihood of positive $\{x_i^+ \mid i = 1,\ldots,N^+\}$ and negative $\{x_i^- \mid i = 1,\ldots,N^-\}$ sequences. The objective function is thus defined as follows:

$$
\begin{aligned}
\mathcal{L}(X^+, X^-, \nu) &= \sum_{i=1}^{N^+} \log \frac{Z^{+}(\nu, x_i^+)}{Z(\nu, x_i^+)} + \sum_{i=1}^{N^-} \log \frac{Z^{-}(\nu, x_i^-)}{Z(\nu, x_i^-)}\\
&\quad - R(\theta,\lambda) \qquad \text{(S19)}\\
Z^{+}(\nu,x) &= \sum_{\tau\in\Phi^{+}(x)} \tilde{P}_{\texttt{couple}}(\tau \mid \nu, x)\\
Z^{-}(\nu,x) &= Z(\nu,x) - Z^{+}(\nu,x)\\
R(\theta,\lambda) &= \rho_\theta\,|\theta|^2 + \rho_\lambda \lambda^2
\end{aligned}
$$

Here, $\Phi^{+}(x)$ represents the set of parse trees, such that a full-length motif occurs somewhere in sequence x. The ratio $Z^{+}(\nu,x)/Z(\nu,x)$ represents the occurrence probability of the motif in x. Then, $Z^{-}(\nu,x)/Z(\nu,x)$ represents the probability of motif absence in x. $R(\theta,\lambda)$ represents a regularization term. We set regularization parameters $\rho_\theta = \rho_\lambda = 0.1$ in all experiments.

The optimal parameters $\hat{\theta}$ and $\hat{\lambda}$ given search pattern μ are obtained by,

$$
\hat{\theta}, \hat{\lambda} = \mathrm{argmax}_{\theta,\lambda} \mathcal{L}(X^+, X^-, \nu) \qquad \text{(S20)}
$$

We optimize parameters based on stochastic gradient descent. First, we obtain the gradient of potential function $\tilde{P}_{\texttt{couple}}(\tau \mid \nu, x)$.

$$
\frac{\partial}{\partial \xi}\tilde{P}_{\texttt{couple}}(\tau \mid \nu, x) = \eta(\tau, x)\tilde{P}_{\texttt{couple}}(\tau \mid \nu, x) \qquad \text{(S21)}
$$

$$
(\xi, \eta) = (\theta_{i,a}, N_{i,a}), (\theta_{i,j,a,b}, N_{i,j,a,b}), (\lambda, \gamma) \qquad \text{(S22)}
$$

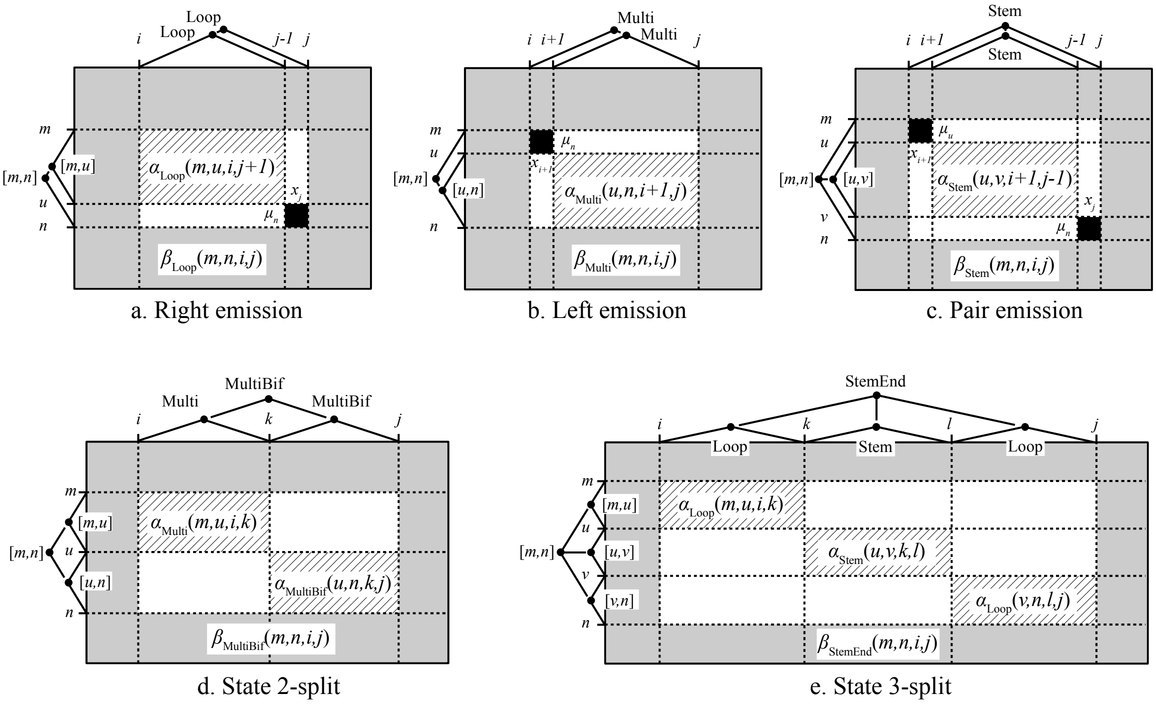

Supplementary Figure S2: **Conceptual diagram of inside outside variable.** The horizontal axis represents the index of the sequence, and the vertical axis represents the index of the search pattern. The shaded block is the range of partial parse trees whose potentials are included in the inside variable, and the grey block represents the outside variable. The black square is location of base/base pair emission. **a** example of right emission, the transition in which `Loop` outputs one base. **b** example of left emission, the transition in which `Multi` outputs one base. **c** example of pair emission, the transition in which `Stem` outputs base pair to both sides. **d** example of state 2-split, the transition in which `MultiBif` branches to `Multi` and `MultiBif` without emission. **e** example of state 3-split, the transition in which `StemEnd` emits an internal loop to both sides and reassemble in `Stem`. This is the only example of a state 3-split in Rfold

We use them to differentiate the partition function and its logarithm.

$$\begin{aligned}\frac{\partial}{\partial \xi} Z(x,\theta,\lambda) &= \sum_{\tau\in\Phi(x)} \frac{\partial}{\partial \xi} \tilde{P}_{\texttt{couple}}(\tau \mid \nu, x)\\ &= \sum_{\tau\in\Phi(x)} \eta(\tau,x)\tilde{P}_{\texttt{couple}}(\tau \mid \nu, x) \qquad \text{(S23)}\end{aligned}$$

$$\begin{aligned}\frac{\partial}{\partial \xi} \log Z(\nu,x) &= \frac{\frac{\partial}{\partial \xi} Z(\nu,x)}{Z(\nu,x)}\\ &= \sum_{\tau\in\Phi(x)} \eta(\tau,x)\frac{\tilde{P}_{\texttt{couple}}(\tau \mid \nu, x)}{Z(\nu,x)}\\ &= \sum_{\tau\in\Phi(x)} \eta(\tau,x)P(\tau \mid \nu, x)\\ &= E\left[\eta(\tau,x)\right] \qquad \text{(S24)}\end{aligned}$$

Similar expressions can be obtained for $Z^{\pm}(\nu,x)$ and for $\log Z^{\pm}(\nu,x)$. Utilizing these equations,

the derivative of the objective function can be expressed as follows.

$$\begin{aligned}\frac{\partial}{\partial \xi}\mathcal{L}(X^+, X^-, \nu) &= \sum_{i=1}^{N^+} E^+ \left[\eta(\tau, x_i^+)\right] - E\left[\eta(\tau, x_i^+)\right] \\ &+ \sum_{i=1}^{N^-} E^- \left[\eta(\tau, x_i^-)\right] - E\left[\eta(\tau, x_i^-)\right] \\ &+ \frac{\partial}{\partial \xi} R(\theta, \lambda)\end{aligned} \tag{S25}$$

where E^+, E^- and E represent the expected values associated with the partition functions Z^+, Z^- and Z, respectively. The expected values $E\left[\eta(\tau, x)\right]$ can be calculated using CFG's inside variable α and outside variable β (Figure S2).

$$E\left[N_{m,a}(\tau, x)\right] = \frac{1}{Z(x, \theta, \lambda)} \Sigma \begin{cases} \text{Right emission} \\ \quad \Pi \begin{cases} \beta_s(u, m, i, j) \\ \tilde{P}_{\texttt{energy}}(\langle s, i, j\rangle \to \langle s', i, j\rangle\, x_j \mid x)^\lambda \\ \tilde{P}_{\texttt{profile}}(\langle [u, m], i, j\rangle \to \langle [u, v], i, j\rangle\, x_j \mid x) \\ \alpha_{s'}(u, v, i, j+1) \end{cases} \\ \text{for } \{u, v \mid [u, m] \to [u, v] x_j \in P_2\} \\ \text{for } \{s, s' \mid s \to s' x_j \in P_1\} \\ \text{for } \{i, j \mid 0 \le i \le j \le |x| \;\&\; x_j = a\} \\ \\ \text{Left emission} \\ \quad \Pi \begin{cases} \beta_s(u, m, i, j) \\ \tilde{P}_{\texttt{energy}}(\langle s, i, j\rangle \to \langle s', i, j\rangle\, x_j \mid x)^\lambda \\ \tilde{P}_{\texttt{profile}}(\langle [u, m], i, j\rangle \to \langle [u, v], i, j\rangle\, x_j \mid x, \theta) \\ \alpha_{s'}(u, v, i, j+1) \end{cases} \\ \text{for } \{u, v \mid [m, v] \to x_i [u, v] \in P_2\} \\ \text{for } \{s, s' \mid s \to x_i s' \in P_1\} \\ \text{for } \{i, j \mid 0 \le i \le j \le |x| \;\&\; x_i = a\} \end{cases} \tag{S26}$$

$$E\left[N_{m,n,a,b}(\tau, x)\right] = \frac{1}{Z(x, \theta, \lambda)} \Sigma \begin{cases} \text{Pair emission} \\ \quad \Pi \begin{cases} \beta_s(u, m, i, j) \\ \tilde{P}_{\texttt{energy}}(\langle s, i, j\rangle \to x_i \,\langle s', i+1, j-1\rangle\, x_j \mid x)^\lambda \\ \tilde{P}_{\texttt{profile}}(\langle [u, m], i, j\rangle \to x_i \,\langle [u, v], i+1, j-1\rangle\, x_j \mid x, \theta) \\ \alpha_{s'}(u, v, i-1, j+1) \end{cases} \\ \text{for } \{u, v \mid [m, n] \to x_i [u, v] x_j \in P_2\} \\ \text{for } \{s, s' \mid s \to x_i s' x_j \in P_2\} \\ \text{for } \{i, j \mid 0 \le i \le j \le |x| \;\&\; x_i = a \;\&\; x_j = b\} \end{cases} \tag{S27}$$

$$
E\left[\gamma(\tau,x)\right] = \frac{1}{Z(x,\theta,\lambda)}\Bigg(
$$

$$
\Sigma\left\{\begin{array}{l}
\text{Right emission} \\
\Pi\left\{\begin{array}{l}
\gamma(\langle s,i,j\rangle \to \langle s',i,j\rangle\, x_j \mid x) \\
\beta_s(u,m,i,j) \\
\tilde{P}_{\text{energy}}(\langle s,i,j\rangle \to \langle s',i,j\rangle\, x_j \mid x)^{\lambda} \\
\tilde{P}_{\texttt{profile}}(\langle [u,m],i,j\rangle \to \langle [u,v],i,j\rangle\, x_j \mid x) \\
\alpha_{s'}(u,v,i,j+1)
\end{array}\right. \\
\text{for } \{u,v \mid [u,m] \to [u,v]x_j \in P_2\} \\
\text{for } \{s,s' \mid s \to s'x_j \in P_1\} \\
\text{for } \{i,j \mid 0 \le i \le j \le |x|\}
\end{array}\right.
+\Sigma\left\{\begin{array}{l}
\text{Left emission} \\
\Pi\left\{\begin{array}{l}
\gamma(\langle s,i,j\rangle \to \langle s',i,j\rangle\, x_j \mid x) \\
\beta_s(u,m,i,j) \\
\tilde{P}_{\text{energy}}(\langle s,i,j\rangle \to \langle s',i,j\rangle\, x_j \mid x)^{\lambda} \\
\tilde{P}_{\texttt{profile}}(\langle [u,m],i,j\rangle \to \langle [u,v],i,j\rangle\, x_j \mid x,\theta) \\
\alpha_{s'}(u,v,i,j+1)
\end{array}\right. \\
\text{for } \{u,v \mid [m,v] \to x_i[u,v] \in P_2\} \\
\text{for } \{s,s' \mid s \to x_i s' \in P_1\} \\
\text{for } \{i,j \mid 0 \le i \le j \le |x|\}
\end{array}\right.
$$

$$
+\Sigma\left\{\begin{array}{l}
\text{Pair emission} \\
\Pi\left\{\begin{array}{l}
\gamma(\langle s,i,j\rangle \to x_i\,\langle s',i+1,j-1\rangle\, x_j \mid x) \\
\beta_s(u,m,i,j) \\
\tilde{P}_{\text{energy}}(\langle s,i,j\rangle \to x_i\,\langle s',i+1,j-1\rangle\, x_j \mid x)^{\lambda} \\
\tilde{P}_{\texttt{profile}}(\langle [u,m],i,j\rangle \to x_i\,\langle [u,v],i+1,j-1\rangle\, x_j \mid x,\theta) \\
\alpha_{s'}(u,v,i-1,j+1)
\end{array}\right. \\
\text{for } \{u,v \mid [m,n] \to x_i[u,v]x_j \in P_2\} \\
\text{for } \{s,s' \mid s \to x_i s' x_j \in P_2\} \\
\text{for } \{i,j \mid 0 \le i \le j \le |x|\}
\end{array}\right.
+\Sigma\left\{\begin{array}{l}
\text{State transition} \\
\Pi\left\{\begin{array}{l}
\gamma(\langle s,i,j\rangle \to \langle s',i,j\rangle \mid x) \\
\beta_s(m,n,i,j) \\
\tilde{P}_{\text{energy}}(\langle s,i,j\rangle \to \langle s',i,j\rangle \mid x)^{\lambda} \\
\alpha_{s'}(m,n,i,j)
\end{array}\right. \\
\text{for } \{m,n \mid [m,n] \to [m,n] \in P_2\} \\
\text{for } \{s,s' \mid s \to s' \in P_1\} \\
\text{for } \{i,j \mid 0 \le i \le j \le |x|\}
\end{array}\right.
$$

$$
+\Sigma\left\{\begin{array}{l}
\text{State 2-split} \\
\Pi\left\{\begin{array}{l}
\gamma(\langle s,i,j\rangle \to \langle s',i,k\rangle\,\langle s'',k,j\rangle \mid x) \\
\beta_s(m,n,i,j) \\
\tilde{P}_{\text{energy}}(\langle s,i,j\rangle \to \langle s',i,k\rangle\,\langle s'',k,j\rangle \mid x)^{\lambda} \\
\alpha_{s'}(m,u,i,k) \\
\alpha_{s''}(u,n,k,l)
\end{array}\right. \\
\text{for } \{m,n,u \mid [m,n] \to [m,u][u,n] \in P_2\} \\
\text{for } \{s,s',s'' \mid s \to s's'' \in P_1\} \\
\text{for } \{i,j,k \mid 0 \le i \le k \le j \le |x|\}
\end{array}\right.
+\Sigma\left\{\begin{array}{l}
\text{State 3-split} \\
\Pi\left\{\begin{array}{l}
\gamma(\langle s,i,j\rangle \to \langle s',i,k\rangle\,\langle s'',k,l\rangle\,\langle s''',l,j\rangle \mid x) \\
\beta_s(m,n,i,j) \\
\tilde{P}_{\text{energy}}(\langle s,i,j\rangle \to \langle s',i,k\rangle\,\langle s'',k,l\rangle\,\langle s''',l,j\rangle \mid x)^{\lambda} \\
\alpha_{s'}(m,u,i,k) \\
\alpha_{s''}(u,v,k,l) \\
\alpha_{s'''}(v,n,l,j)
\end{array}\right. \\
\text{for } \{m,n,u,v \mid [m,n] \to [m,u][u,v][v,n] \in P_2\} \\
\text{for } \{s,s',s'',s''' \mid s \to s's''s''' \in P_1\} \\
\text{for } \{i,j,k,l \mid 0 \le i \le k \le l \le j \le |x|\}
\end{array}\right.
$$

$$
\Bigg) \tag{S28}
$$

$\alpha_v(m,n,i,j)$ and $\beta_v(m,n,i,j)$ are the inside outside variables that corresponds to the RNAelem hidden variable $(v,[m,n]) \in V$ spanning the arrays $x_i \cdots x_{j-1}$. The inside outside variable is the sum of the potential upstream and downstream of the CFG state across the entire parse trees. To calculate α and β exactly, the inside and outside algorithms are used.

for $\{i \mid 0 \le i \le |x|\}$

$$
\alpha_{\texttt{Loop}}(m,n,i,j) = \Sigma\left\{\begin{array}{l}
\Pi\left\{\begin{array}{l}
\alpha_{\texttt{Loop}}(m,u,i,j-1) \\
\tilde{P}_{\text{energy}}(\langle \texttt{Loop},i,j\rangle \to \langle \texttt{Loop},i,j-1\rangle\, x_{j-1} \mid x)^{\lambda} \\
\tilde{P}_{\texttt{profile}}(\langle [m,n],i,j\rangle \to \langle [m,u],i,j-1\rangle\, x_{j-1} \mid x,\theta)
\end{array}\right. \\
\text{for } \{u \mid [m,n] \to [m,u]x_{j-1} \in P_2\}
\end{array}\right. \tag{S29}
$$

$$
\alpha_{\texttt{MultiBif}}(m,n,i,j) = \Sigma\left\{\begin{array}{l}
\Pi\left\{\begin{array}{l}
\alpha_{\texttt{Multi1}}(m,u,i,k) \\
\alpha_{\texttt{Multi2}}(u,n,k,j) \\
\tilde{P}_{\text{energy}}(\langle \texttt{MultiBif},i,j\rangle \to \langle \texttt{Multi1},i,k\rangle\,\langle \texttt{Multi2},k,j\rangle \mid x)^{\lambda}
\end{array}\right. \\
\text{for } \{u \mid [m,n] \to [m,u][u,n] \in P_2\} \\
\text{for } \{k \mid i < k < j\}
\end{array}\right. \tag{S30}
$$

$$
\alpha_{\texttt{Multi2}}(m,n,i,j) = \Sigma\left\{\begin{array}{l}
\Pi\left\{\begin{array}{l}
\alpha_{\texttt{Stem}}(m,n,i,j) \\
\tilde{P}_{\text{energy}}(\langle \texttt{Multi2},i,j\rangle \to \langle \texttt{Stem},i,j\rangle \mid x)^{\lambda}
\end{array}\right. \\
\Pi\left\{\begin{array}{l}
\alpha_{\texttt{Multi2}}(m,u,i,j-1) \\
\tilde{P}_{\text{energy}}(\langle \texttt{Multi2},i,j\rangle \to \langle \texttt{Multi2},i,j-1\rangle\, x_{j-1} \mid x)^{\lambda} \\
\tilde{P}_{\texttt{profile}}(\langle [m,n],i,j\rangle \to \langle [m,u],i,j-1\rangle\, x_{j-1} \mid x,\theta)
\end{array}\right. \\
\text{for } \{u \mid [m,n] \to [m,u]x_{j-1} \in P_2\}
\end{array}\right. \tag{S31}
$$

$$
\alpha_{\texttt{Multi1}}(m,n,i,j) = \Sigma\left\{\begin{array}{l}
\Pi\left\{\begin{array}{l}
\alpha_{\texttt{Multi2}}(m,n,i,j) \\
\tilde{P}_{\text{energy}}(\langle \texttt{Multi1},i,j\rangle \to \langle \texttt{Multi2},i,j\rangle \mid x)^{\lambda}
\end{array}\right. \\
\Pi\left\{\begin{array}{l}
\alpha_{\texttt{MultiBif}}(m,n,i,j) \\
\tilde{P}_{\text{energy}}(\langle \texttt{Multi1},i,j\rangle \to \langle \texttt{MultiBif},i,j\rangle \mid x)^{\lambda}
\end{array}\right.
\end{array}\right. \tag{S32}
$$

$$
\alpha_{\texttt{Multi}}(m,n,i,j) = \Sigma \begin{cases} \Pi \begin{cases} \alpha_{\texttt{Multi}}(u,n,i+1,j) \\ \tilde{P}_{\texttt{energy}}(\langle \texttt{Multi}, i, j\rangle \to x_i \, \langle \texttt{Multi}, i+1, j\rangle \mid x)^{\lambda} \\ \tilde{P}_{\texttt{profile}}(\langle [m,n], i, j\rangle \to x_i \, \langle [u,n], i+1, j\rangle \mid x, \theta) \end{cases} \\ \text{for } \{u \mid [m,n] \to x_i[u,n] \in P_2\} \\ \Pi \begin{cases} \alpha_{\texttt{MultiBif}}(m,n,i,j) \\ \tilde{P}_{\texttt{energy}}(\langle \texttt{Multi}, i, j\rangle \to \langle \texttt{MultiBif}, i, j\rangle \mid x)^{\lambda} \end{cases} \end{cases} \tag{S33}
$$

$$
\alpha_{\texttt{Stem}}(m,n,i,j) = \Sigma \begin{cases} \Pi \begin{cases} \alpha_{\texttt{Stem}}(u,v,i+1,j-1) \\ \tilde{P}_{\texttt{energy}}(\langle \texttt{Stem}, i, j\rangle \to x_{i+1} \, \langle \texttt{Stem}, i+1, j-1\rangle \, x_j \mid x)^{\lambda} \\ \tilde{P}_{\texttt{profile}}(\langle [m,n], i, j\rangle \to x_i \, \langle [u,v], i+1, j-1\rangle \, x_j \mid x, \theta) \end{cases} \\ \text{for } \{u, v \mid [m,n] \to x_{i+1}[u,v]x_j \in P_2\} \\ \Pi \begin{cases} \alpha_{\texttt{StemEnd}}(u,v,i,j) \\ \tilde{P}_{\texttt{energy}}(\langle \texttt{Stem}, i, j\rangle \to x_{i+1} \, \langle \texttt{StemEnd}, i+1, j-1\rangle \, x_j \mid x)^{\lambda} \\ \tilde{P}_{\texttt{profile}}(\langle [m,n], i, j\rangle \to x_i \, \langle [u,v], i+1, j-1\rangle \, x_j \mid x, \theta) \end{cases} \\ \text{for } \{u, v \mid [m,n] \to x_{i+1}[u,v]x_j \in P_2\} \end{cases} \tag{S34}
$$

$$
\alpha_{\texttt{StemEnd}}(m,n,i,j) = \Sigma \begin{cases} \Pi \begin{cases} \alpha_{\texttt{Loop}}(m,n,i,j) \\ \tilde{P}_{\texttt{energy}}(\langle \texttt{StemEnd}, i, j\rangle \to \langle \texttt{Loop}, i, j\rangle \mid x)^{\lambda} \end{cases} \\ \text{if } [m,n] \in V_2^{\texttt{Loop}} \\ \Pi \begin{cases} \alpha_{\texttt{Loop}}(m,u,i,k) \\ \alpha_{\texttt{Stem}}(u,v,k,l) \\ \alpha_{\texttt{Loop}}(v,n,l,j) \\ \tilde{P}_{\texttt{energy}}(\langle \texttt{StemEnd}, i, j\rangle \to \langle \texttt{Loop}, i, k\rangle \, \langle \texttt{Stem}, k, l\rangle \, \langle \texttt{Loop}, l, j\rangle \mid x)^{\lambda} \end{cases} \\ \text{for } \{u, v \mid [m,n] \to [m,u][u,v][v,n] \in P_2\} \\ \text{for } \{k, l \mid i \le k < l \le j, 0 < (k-i) + (j-l) < \texttt{MaxIntLoop}\} \end{cases} \tag{S35}
$$

$$
\alpha_{\texttt{Outer}}(0,n,0,j) = \Sigma \begin{cases} \Pi \begin{cases} \alpha_{\texttt{Outer}}(0,u,0,j-1) \\ \tilde{P}_{\texttt{energy}}(\langle \texttt{Outer}, 0, j\rangle \to \langle \texttt{Outer}, 0, j-1\rangle \, x_{j-1} \mid x)^{\lambda} \\ \tilde{P}_{\texttt{profile}}(\langle [m,n], 0, j\rangle \to \langle [m,u], 0, j-1\rangle \, x_{j-1} \mid x, \theta) \end{cases} \\ \text{for } \{u \mid [0,n] \to [0,u]x_{j-1} \in P_2\} \\ \Pi \begin{cases} \alpha_{\texttt{Outer}}(0,u,0,k) \\ \alpha_{\texttt{Stem}}(u,n,k,j) \\ \tilde{P}_{\texttt{energy}}(\langle \texttt{Outer}, 0, j\rangle \to \langle \texttt{Outer}, 0, k\rangle \, \langle \texttt{Stem}, k, j\rangle \mid x)^{\lambda} \end{cases} \\ \text{for } \{u \mid [0,n] \to [0,u][u,n] \in P_2\} \\ \text{for } \{k \mid k < j\} \end{cases} \tag{S36}
$$

where MaxIntLoop denotes the maximum length of an internal loop, including bulge loops. This parameter is consistently set at 30 in this study. $V_2^{\texttt{Loop}}$ forms a subset of V_2 with interval states that can emit consecutive loop regions, or formally

$$
V_2^{\texttt{Loop}} = \{[m,n] | m \le n, D_m = D_{m+1} = \cdots = D_n\} \tag{S37}
$$

with scope index D obtained by Algorithm S1. $\tilde{P}_{\texttt{energy}}$ represents the potential of the Rfold model given by Equation S1. $\tilde{P}_{\texttt{profile}}$ represents the potential of Profile CFG given by Equation S7.

The computational complexity for parameter fitting is dominated by the inside/outside algorithm. This dynamic programming technique exhibits a computational complexity of $\mathcal{O}(NLW^2|P|)$, where N is the number of sequences, L is the length of each sequence, W is the maximal span, and $|P|$ represents the number of state transition rules in the coupled CFG. In the coupled CFG, quantity $|P|$ can be estimated by using the number of ways of coupling the interval states of the Profile CFG with each of the constant state transition rules of the Rfold model. The Profile CFG possesses $\mathcal{O}(|\mu|^2)$ interval states. Furthermore, accounting for degrees of freedom in state branching, it is estimated that $\mathcal{O}(|P|) = \mathcal{O}(|\mu|^4)$. Performance evaluation within our environment are detailed in the section 1.10: “Execution time”.

These algorithms for the coupled CFG are based on Sankoff’s method, which has solved the structural alignments of two RNA sequences using two instances of a single CFG (Sankoff, 1985). We explained above that the hidden variable $v_1 \in V_1$ of Rfold and the hidden variable $v_2 \in V_2$ of Profile CFG simultaneously span the region $x_i \cdots x_j$ of RNA sequence x. On the other hand, we can also view that the hidden variable $v \in V$ of RNAelem spans a rectangular region $x_i \cdots x_j$ of the

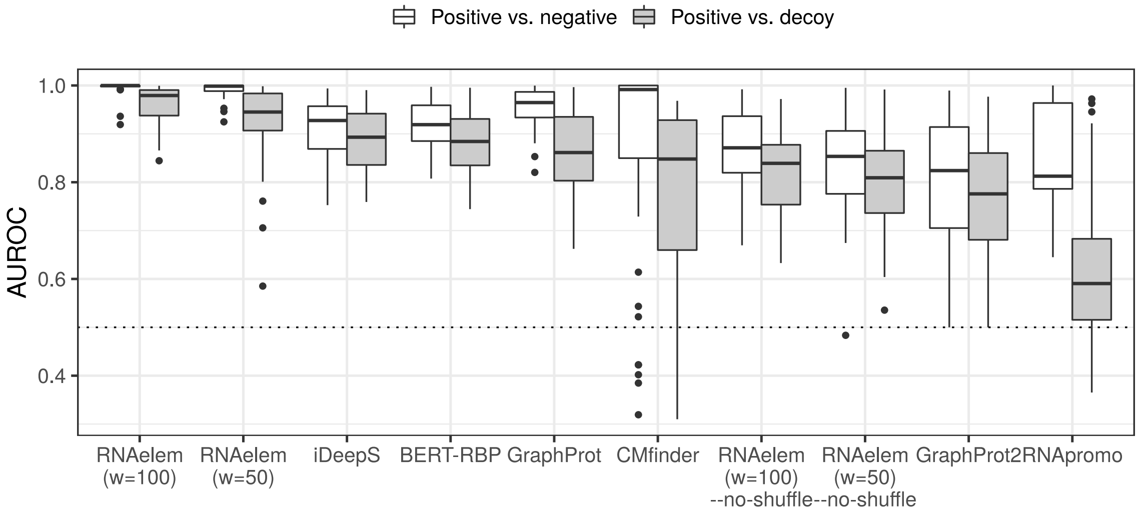

Supplementary Figure S3: **Impact in AUROC of the simulated negative sequences technique when fitting parameters.** AUROC for identifying simulated positive, negative, and decoy sequences are compared among tools. RNAelem uses a simulated negative sequences with shuffling that conserves the frequency of 2-mer occurences when fitting parameters. AUROC with that feature turned off is shown as `--no-shuffle`.

RNA sequence x and region $\mu_m \cdots \mu_n$ of the search pattern μ. This interpretation relates RNAelem to the Sankoff algorithm, as the model underlying the Sankoff algorithm can be considered as the coupled CFG of two Rfold models and its hidden state spans rectangular region $x_i \cdots x_j$ and $y_i \cdots y_j$ of two RNA sequences x and y.

1.5 Methods for stabilizing gradient-based optimization

In gradient-based optimization of multivariable function, it is a general problem to trap to a local optimum. A direct solution to this problem is to increase the size of data set. While it is impossible to generate a positive sequence, it is easy to generate practical negative sequences through shuffling. Therefore, we generated simulated negatives through shuffling positive sequences while maintaining the frequency of 2-mer occurrences. This technique has the advantage that many negative sequences can be generated for a single positive sequence. An ablation study found that this practice significantly improves the AUROC (Figure S3)

Stochastic optimization is another technique that can be used to improve convergence rate in multivariable function optimization. In this study, Adam (Kingma and Ba, 2014) was used. In particular, positive and negative sequences were divided into batches of a default size of 64. For each iteration, 64 positive, 64 negative, and 64 simulated negative sequences were used to evaluate the derivatives of the objective function. We used step size $\alpha = 0.001$, exponential decay rate $\beta_1 = 0.9$, $\beta_2 = 0.999$ and step size auxiliary parameter $\epsilon = 10^{-8}$ as hyper parameters.

Furthermore, a significant challenge in training stochastic models is their sensitivity to initial values. To mitigate this, the renowned sequence motif search tool, MEME, introduced a feature to allocate a position specific prior (PSP) to motif occurrences (Bailey et al., 2009). Inspired by this, we first detected short sequence fragments that appear significantly in positive sequences and assigned PSPs based on their occurrence locations. This approach substantially enhanced the stability of parameter fitting. First, the PSP for each position in the sequences $x^{(1)}, \ldots, x^{(N)}$ of all positive sets is initialized with $w_i^{(n)} = 1$. For each k-mer of $k = k_{\min}, \ldots, k_{\max}$, the Fisher-test determines which k-mer is enriched in the positive set that is most significantly compared to the negative set.

Supplementary Algorithm S3 Enumerate secondary structures for given length.

```
procedure RECURSION(structure, remainingLength, currentDepth)
    if remainingLength == 0 then
        if currentDepth == 0 then
            if "()" or "(.)" or "(..)" or ")(" in structure then
                RETURN
            else
                PRINT(structure)                                ▷ Print the secondary structure
            end if
        end if
        RETURN
    end if
    CALL RECURSION(structure + ".", remainingLength −1, currentDepth)
    CALL RECURSION(structure + "(", remainingLength −1, currentDepth +1)
    if currentDepth > 0 then
        CALL RECURSION(structure + ")", remainingLength −1, currentDepth −1)
    end if
end procedure
CALL RECURSION("", length, 0)
```

For all k-mers of the same size that have less than p-value of 0.05, the w_i of the region of length k where the k-mer appeared is used by κ. In this study, we used $k_{\min} = 3, k_{\max} = 10, \kappa = 0.1$ in all experiments. Finally, we replace the alignment function in Profile CFG with the following $\tilde{P}^{\dagger}_{\texttt{profile}}$:

$$
\begin{aligned}
\tilde{P}^{\dagger}_{\texttt{profile}}(\phi \mid x, \theta, w) &= \prod_{p \in \phi} \tilde{P}^{\dagger}_{\texttt{profile}}(p \mid \theta, w) \\
&= \prod_{p \in \phi} \tilde{P}_{\texttt{profile}}(p \mid \theta) \begin{cases} w_i, & \text{if } p = \langle [m,n], i, j \rangle \to x_i \, \langle [u,n], i+1, j \rangle \\ w_j, & \text{if } p = \langle [m,n], i, j \rangle \to \langle [m,v], i, j-1 \rangle \, x_j \\ w_i w_j, & \text{if } p = \langle [m,n], i, j \rangle \to x_i \, \langle [u,v], i+1, j-1 \rangle \, x_j \\ 1, & \text{otherwise} \end{cases}
\end{aligned}
\tag{S38}
$$

1.6 Candidate patterns and model selection

There is very little knowledge about secondary structure motifs in RBP-binding sites, and there are no large-scale experimental datasets. Therefore, we created a search pattern set that covers the theoretical secondary structure space as comprehensively as possible within the range that avoids computational explosion

We used a fixed set of search patterns in all experiments (Table S1); for a total of 135 patterns including (1) a loop structure with a length of 3 to 9, (2) all possible secondary structure patterns with a length of 10, which is enumerated by Algorithm S3, and (3) patterns with a gap inserted at the center of each of (2). For each search pattern, RNAelem optimizes the parameters θ and λ.

Theoretically, an insertion region could be placed between any two symbols of the enumerated search pattern, which would increase the number of search patterns by 2^9, rendering computation infeasible. Therefore, in this study, we limited the insertion region to a single central location within the pattern to maximize the motif model's flexibility.

In practice, the candidate search patterns can be customized by the user. Typically, one can narrow down the domain of candidates (e.g., only hairpin structures) using prior knowledge obtained from external experiments. Conversely, one can also conduct broader searches including longer search pattern candidates, by leveraging more extensive computational resources.

Supplementary Algorithm S4 Computing Enrichment Score (ES)

procedure ComputeES
  Input:
    Positive sequence set X^+
    Negative sequence set X^-
    Motif model $\nu = (\mu, \theta, \lambda)$
  Output:
    Enrichment Score (ES) $\in \mathbb{R}$
  Initialize:
    True positives $(TP) \leftarrow |X^+|$
    False positives $(FP) \leftarrow |X^-|$
    True negatives $(TN) \leftarrow 0$
    False negatives $(FN) \leftarrow 0$
    Previous False Positive Rate $(FPR') \leftarrow 1$
    Previous True Positive Rate $(TPR') \leftarrow 1$
    Area Under ROC Curve $(AUROC) \leftarrow 0$
  Apply motif model ν to calculate the score $P(y = 1 \mid \nu, x)$ for each sequence x in X^+ and X^-
  Create tuples (s_i, p_i) for each sequence, where s_i is the score, $p_i \in \{0, 1\}$ is positive flag
  Sort the tuples based on scores s_i
  for each tuple (s_i, p_i) **do**
    $TP \leftarrow TP - p_i$
    $FN \leftarrow FN + p_i$
    $FP \leftarrow FP - (1 - p_i)$
    $TN \leftarrow TN + (1 - p_i)$
    False Positive Rate $(FPR) \leftarrow \frac{FP}{TN+FP}$
    True Positive Rate $(TPR) \leftarrow \frac{TP}{TP+FN}$
    $AUROC \leftarrow AUROC + \frac{(FPR' - FPR) \times (TPR' + TPR)}{2}$
    $FPR' \leftarrow FPR$, $TPR' \leftarrow TPR$
  end for
  $ES \leftarrow AUROC + \frac{FPR' \times TPR'}{2}$
  Return ES
end procedure

Supplementary Table S1: **Search pattern pools used in all experiments in this study.** We enumerated the following 135 search patterns: (1) loop structures of lengths 3 to 9, (2) all possible secondary structures of length 10, and (3) patterns with only one gap inserted in the middle of (2)

1	`*...*`	`*....*`	`*.....*`	`*......*`
5	`*.......*`	`*........*`	`*.........*`	`*..........*`
9	`*(...).....*`	`*(....)....*`	`*(.....)...*`	`*(......)..*`
13	`*(.......).*`	`*(........)*`	`*.(...)....*`	`*.(....)...*`
17	`*.(.....)..*`	`*.(......).*`	`*.(.......)*`	`*..(...)...*`
21	`*..(....)..*`	`*..(.....).*`	`*..(......)*`	`*...(...)..*`
25	`*...(....).*`	`*...(.....)*`	`*....(...).*`	`*....(....)*`
29	`*.....(...)*`	`*((...))...*`	`*((...).)..*`	`*((...)..).*`
33	`*((...)...)*`	`*((....))..*`	`*((....).).*`	`*((....)..)*`
37	`*((.....)).*`	`*((.....).)*`	`*((......))*`	`*(.(...))..*`
41	`*(.(...).).*`	`*(.(...)..)*`	`*(.(....)).*`	`*(.(....).)*`
45	`*(.(.....))*`	`*(..(...)).*`	`*(..(...).)*`	`*(..(....))*`
49	`*(...(...))*`	`*.((...))..*`	`*.((...).).*`	`*.((...)..)*`
53	`*.((....)).*`	`*.((....).)*`	`*.((.....))*`	`*.(.(...)).*`
57	`*.(.(...).)*`	`*.(.(....))*`	`*.(..(...))*`	`*..((...)).*`
61	`*..((...).)*`	`*..((....))*`	`*..(.(...))*`	`*...((...))*`
65	`*(((...))).*`	`*(((...)).)*`	`*(((...).))*`	`*(((....)))*`
69	`*((.(...)))*`	`*(.((...)))*`	`*.(((...)))*`	`*.....*.....*`
73	`*(...)*.....*`	`*(....*)....*`	`*(....*.)...*`	`*(....*..)..*`
77	`*(....*...).*`	`*(....*....)*`	`*.(...*)....*`	`*.(...*.)...*`
81	`*.(...*..)..*`	`*.(...*...).*`	`*.(...*....)*`	`*..(..*.)...*`
85	`*..(..*..)..*`	`*..(..*...).*`	`*..(..*....)*`	`*...(.*..)..*`
89	`*...(.*...).*`	`*...(.*....)*`	`*....(*...).*`	`*....(*....)*`
93	`*.....*(...)*`	`*((...*))...*`	`*((...*).)..*`	`*((...*)..).*`
97	`*((...*)...)*`	`*((...*.))..*`	`*((...*.).).*`	`*((...*.)..)*`
101	`*((...*..)).*`	`*((...*..).)*`	`*((...*...))*`	`*(.(..*.))..`
105	`*(.(..*.).).*`	`*(.(..*.)..)*`	`*(.(..*..)).*`	`*(.(..*..).)*`
109	`*(.(..*...))*`	`*(..(.*..)).*`	`*(..(.*..).)*`	`*(..(.*...))*`
113	`*(...(*...))*`	`*.((..*.))..*`	`*.((..*.).).*`	`*.((..*.)..)*`
117	`*.((..*..)).*`	`*.((..*..).)*`	`*.((..*...))*`	`*.(.(.*..)).*`
121	`*.(.(.*..).)*`	`*.(.(.*...))*`	`*.(..(*...))*`	`*..((.*..)).*`
125	`*..((.*..).)*`	`*..((.*...))*`	`*..(.(*...))*`	`*...((*...))*`
129	`*(((..*.))).*`	`*(((..*.)).)*`	`*(((..*.).))*`	`*(((..*..)))*`
133	`*((.(.*..)))*`	`*(.((.*..)))*`	`*.(((.*..)))*`	

1.7 Computing Enrichment Score (ES)

The trained Coupled CFGs $\hat{\nu}$ are compared based on their significance. In our study, significance of motifs is defined using the Enrichment Score (ES), which is assessed using the Area Under the Receiver Operating Characteristic (AUROC) curve obtained through two-fold cross-validation. Each trained Coupled CFG $\hat{\nu}$ is applied to both positive and negative validation sequences to evaluate the motif occurrence probability $P(y = 1 \mid \hat{\nu}, x)$. This score is used for discriminating positive set from negative sets and the corresponding false positive rate (FPR) and true positive rate (TPR) is plotted varying the decision threshold. Then, AUROC is obtained by accumulating piecewise area under the ROC curve. The method for computing ES is detailed in Supplementary Algorithm S4. The ES, which range from approximately 0.5 to a maximum of 1.0, indicate the motif model's accuracy in distinguishing between positive and negative sets. Higher values indicate motif enrichment exclusively in the positive set. This metric enables non-parametric comparison across patterns of varying lengths and structures. That is, shorter, simpler patterns often have higher ES due to clear sequence-structure conservation, while longer patterns may have lower ES due to overfitting. This

assists in selecting models with complexity suitable for the dataset. Additionally, it was confirmed that this model selection approach using ES effectively mitigates the risk of false positive motif detection (Supplementary Figure S8).

1.8 Procedure from input to output

Supplementary Algorithm S5 Overall algorithm for motif discovery: training and selection of Coupled CFG.

procedure DiscoverMotif
  Input:
    Positive sequence set X^+
    Negative sequence set X^-
    Search pattern set $\mathcal{P}$
  Output:
    Optimal motif model $\hat{\nu}_{\max}$
  Motif model set $\mathcal{N} \leftarrow \emptyset$
  for each search pattern $\mu \in \mathcal{P}$ **do**
    $\mu, \theta, \lambda \leftarrow$ ConstructCoupledCFG (μ)
    Motif model $\nu \leftarrow (\mu, \theta, \lambda)$
    $\mathcal{N} \leftarrow \mathcal{N} \cup \{\nu\}$
  end for
  for each motif model $\nu \in \mathcal{N}$ **do**
    Sum of Enrichment Score (ES) $\alpha \leftarrow 0$
    for each fold k in cross validation **do**
      $X_k^+, X_k^- \leftarrow$ Training set for k-th fold
      $\hat{\nu}_k \leftarrow$ TrainCoupledCFG (X_k^+, X_k^-, ν)
      $\alpha \leftarrow \alpha +$ ComputeES $(X^+ \setminus X_k^+, X^- \setminus X_k^-, \hat{\nu}_k)$
    end for
    $ES[\nu] \leftarrow \alpha$
  end for
  $\nu_{\max} \leftarrow \arg_\nu \max ES[\nu]$
  $\hat{\nu}_{\max} \leftarrow$ TrainCoupledCFG $(X^+, X^-, \nu_{\max})$
  Return $\hat{\nu}_{\max}, ES[\nu_{\max}]$
end procedure

Training phase: In the training phase, the input comprises a set $\mathcal{P}$ of search patterns, a set $X^+ = \{x_1^+, \dots, x_{N^+}^+\}$ of positive sequences, and a set $X^- = \{x_1^-, \dots, x_{N^-}^-\}$ of negative sequences. Search patterns $\mathcal{P}$, defined in dot-bracket notation with asterisks as detailed in the Supplementary section "Definition of search pattern", are either user-provided based on specific interests or generated exhaustively by RNAelem for a given motif size (Supplementary Algorithm S3). Positive sequences X^+ are typically unaligned RNA sequences found in CLIP-seq data peak regions. The negative sequences X^- are either sampled from non-peak regions of CLIP-seq data or are artificially shuffled random sequences.

Upon receiving the inputs, RNAelem begins by transforming each pattern $\mu \in \mathcal{P}$ into a corresponding Coupled CFG $\nu \in \mathcal{N}$, detailed in the "Construction of Coupled CFG" section. A Coupled CFG is defined by the tuple $\nu = (\mu, \theta, \lambda)$, which includes the search pattern μ, the base and base-pair profile θ, and a scaling factor λ. A K-fold cross-validation approach is employed with the iterative training of each Coupled CFG ν, partitioning the data into training sets X_k^+ and X_k^- for k-th fold, and fitting each Coupled CFG $\nu \in \mathcal{N}$ to these subsets. This process is elaborated in the "Training of Coupled CFG" section.

For each Coupled CFG $\hat{\nu}_k = (\mu, \hat{\theta}_k, \hat{\lambda}_k)$ with trained parameters in the k-th round of cross validation, the Enrichment Score (ES) is calculated to evaluate the significance of the motif model. ES, defined as the Area Under the Receiver Operating Characteristic (AUROC) curve, represents

the accuracy of discriminating between positive and negative subsets of sequences which are not used for training the parameters. Further details on this computation are provided in the Supplementary section “Computing Enrichment Score (ES)”.

The Coupled CFGs ν are then ranked based on their ES. Then, we select the Coupled CFG $\nu_{\max}$ with the highest ES. This selected model $\nu_{\max}$ undergoes a final round of training using the complete sets of X^+ and X^- to refine its parameters. RNAelem's output is this final motif model $\hat{\nu}_{\max}$, accompanied by its corresponding ES.

Search phase: In the search phase, the process involves applying a motif model to new test sequences. The inputs to this phase are a trained Coupled CFG $\hat{\nu}$, and a set of sequences $X = \{x_1, \ldots, x_N\}$. The outputs of this phase include the following components:

1. The probability of each sequence containing the motif, expressed as $P(y_i = 1 \mid \hat{\nu}, x_i)$, where the presence and absence of the motif in sequence x_i are represented by $y_i = 1$ and $y_i = 0$, respectively.

2. The most likely parse tree for each sequence, formulated as $\arg_{\tau_i} \max P(\tau_i \mid \hat{\nu}, x_i)$, representing the most probable structural interpretation of the sequence under the given CFG.

3. The probability that each position within each sequence is part of the motif region, denoted as $P(z_{i,j} = 1 \mid \hat{\nu}, x_i)$ for $1 \leq j \leq L_i$, where L_i is the length of the sequence x_i. Here, $z_{i,j} = 1$ indicates that position j is in the motif region, and $z_{i,j} = 0$ indicates its inclusion in the background region.

This framework enables a comprehensive analysis of sequences for motif presence, specific location within the sequence, and the structural configuration that best explains the observed sequence data in the context of the trained motif model.

1.9 Model interpretability of RNAelem

We designed RNAelem to minimize the inclusion of black-box components within the model. The motif logo generated by RNAelem not only visualizes the predicted motifs but also comprehensively represents the model structure of the learned profile CFG. This representation is complete except for a single parameter of state self-transition. Moreover, the annotated search pattern directly embodies the state transition rules of the CFG, and the size of each character (or character pair) in the sequence logo is proportionate to the numerical value of the corresponding parameter.

The likelihood function employed by RNAelem is a straightforward product of the Boltzmann factors of the Profile CFG and the Turner energy model, excluding the parameter λ, which balances the stability of the secondary structure against the strength of the motif match. While the Turner energy model itself is intricate, containing over 10,000 experimentally determined parameters, its behavior has been extensively studied and understood over the past three decades.

Parameter optimization in RNAelem is specifically designed to maximize the likelihood ratio, ensuring motifs are identified in positive samples while absent in negatives. The significance of the estimated motifs is determined using the AUROC score, a metric with low model dependency and well-recognized among data scientists. This strategic design choice enhances the interpretability of RNAelem's model structure and each parameter, offering clear advantages over models based on deep neural networks (DNN) or support vector machines (SVM), which typically operate as more opaque systems.

1.10 Execution time

Our methodology involves the computation of base and base-pair profiles on the search pattern through iterative optimization. Here, each iteration calculates the gradient of the parameters, folding in the full dynamics of the secondary structure model through dynamic programming. This component constitutes the most computationally intensive part of the process.

The computational complexity of the dynamic programming is $\mathcal{O}(NLW^2|P|)$, where N represents the number of sequences, L represents the length of each sequence, W represents the maximal span,

and $|P|$ represents the number of state transition rules in the coupled CFG. Since each search pattern is independently processed, the time required when a de novo search is performed is proportional to the number of search patterns. Thus, if the target structure families, such as hairpin or loop structures, can be restricted, computational time is considerably reduced.

We observed the computational performance of our approach under a test setup with 64 sequences, each having a length 100, a maximal span of 50, and a search pattern length of 10. When executed on a Xeon Gold 6154 (3.0 GHz) CPU, the average processing time per iteration was 5.63 seconds. When the maximal span was increased to 100 under the same conditions, the average processing time extended to 13.5 seconds.

Iterations are carried out until the loss descent rate converges to 10^{-5} or terminates at a maximum of 300 iterations. Under these settings, it takes 1,689 seconds per search pattern with a maximal span of 50 and 4,050 seconds with a maximal span of 100.

2 Data preparation

2.1 Preparation of simulated dataset

We generated a set of sequences with embedded structural motifs using the external tool antaRNA, which outputs an RNA sequence for a given target secondary structure (Kleinkauf et al., 2015). antaRNA incorporates a fuzzy constraints feature, which specifies only local structures. This feature allows structural motifs to be simulated. Structural motifs were randomly selected from among local secondary structures of length 10, and gaps were inserted in the middle. The length of the gap varied between 1 and 10 per sequence. antaRNA accepts ambiguous IUPAC base code to partially specify the sequence in the fuzzy-constrained region. This feature was used to generate conserved sequences with specific structures. antaRNA was also used to generate negative sequences through not specifying the target structure.

Subsequently, data curation was performed to ensure that the generated sequences exhibited the expected structures. Using the Vienna RNA package (Lorenz et al., 2011), we computed the loop probability for each position in the GT motifs and the base-pairing probability for every pair of positions in the GT motifs. Structural motifs with a loop probability of at least 0.5 in the loop region and a base-pairing probability of at least 0.5 in the stem region were classified as positive examples. Formally, all sequences in each dataset had motifs embedded with more than or equal to 0.5 structure consistency, defined as:

$$\text{Structure consistency} = \min \begin{cases} \min_{i \in \text{loop}} \left(1 - \sum_j p_{ij}\right) \\ \min_{i,j \in \text{stem}} p_{ij} \end{cases} \tag{S39}$$

This process yielded 43 structural motifs, each represented by at least 200 samples. The sequences and structural motifs for each dataset are summarized in Table S2, with half of the samples used for training and the remainder for testing. Additionally, a decoy dataset containing only primary sequence motifs was generated to ascertain whether the model could distinguish between sequence and structural information. The motif regions were randomly relocated within the host sequence.

Figure S4 illustrates the distribution of the simulated dataset. The X-axis represents sequence conservation using Shannon entropy, and the Y-axis represents structural consistency. The plot shows that the X-coordinates of each dataset range from 6 to 20, where 6 indicates three conserved bases and 20 indicates ten conserved bases, reflecting the characteristic length of RBP binding motifs. The Y-coordinates range from 0.5 to 1.0, suggesting that at any given time, the desired secondary structure is formed with a probability between 0.5 and 1.0. These features reflect the sequence conservation level and thermodynamic fluctuations of the RNA targeted by RBPs. The Shannon entropy averages 14.6 with a variance of 22.6, while the structural consistency averages 0.689 with a variance of 0.0118.

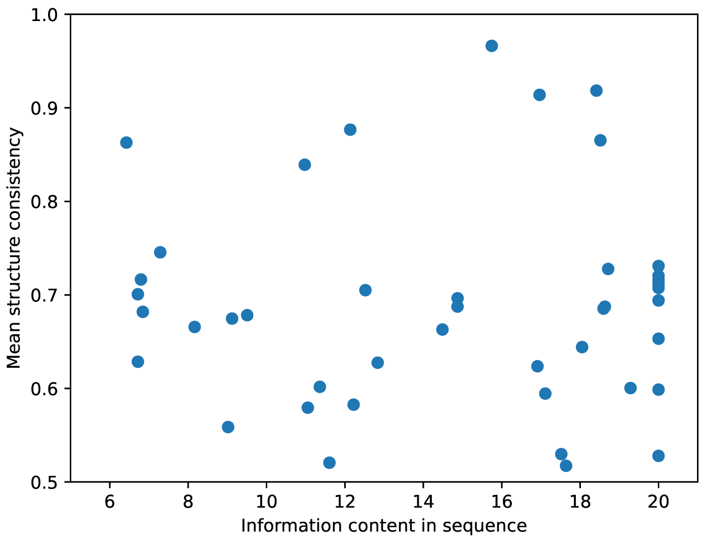

Supplementary Figure S4: **Distribution of simulated dataset** Plotting information content and secondary structure consistency across datasets.

2.2 Prepartion of biological dataset

For biological sequences, data from eCLIP-Seq (Van Nostrand et al., 2016) were used. Generally, CLIP-Seq peaks contain many false positives. eCLIP-Seq is one of a few CLIP-Seq variants that mitigates this problem, in this case by sequencing RNA fragments in their pre-immunoprecipitation state and using them as negative controls (size-matched input technique, or SMInput). This high purity of the eCLIP-Seq peaks made it suitable for use in inferring structural motifs.

The eCLIP database that is hosted by the ENCODE project contains 167 human RBP-binding proteins (Van Nostrand et al., 2020). All of its peak data were downloaded in the narrow BED format. To reduce noise, only intersect regions with multiple replicates and biosamples were used. Then, sequences with top-1000 median peak scores were considered curated peaks. Because the secondary structure in a peak depends on the surrounding sequence, peaks with length that were shorter than 100 were extended equally on both sides to reach length 100. We split each dataset into two equal-size training set and validation set. Negative example sequences were randomly drawn from the transcribed regions of genes having at least one peak, excluding the peak regions.

RNAelem internally generates negative sequences for use in both training of the Coupled CFG and ES calculation (simulated negative), along with the given negative set. These simulated negative sequences are produced using uShuffle, a tool that randomly shuffles sequences while maintaining their 2-mer frequency (Jiang et al., 2008). For training the Coupled CFG, batches of simulated negatives of the same size as batches of positive sequences are used in each iteration. For ES calculation, an equal number of simulated negatives as the entire positive set are used.

2.3 Comparison with other tools

We compared six representative tools that can be used for detecting RNA binding motifs as RNAelem: CMfinder (Yao et al., 2006), RNApromo (Rabani et al., 2008), GraphProt (Maticzka et al., 2014), iDeepS (Pan et al., 2018), BERT-RBP (Yamada and Hamada, 2022), and GraphProt2 (Uhl et al., 2021). CMfinder and RNApromo are based on Profile CFG and are capable of outputting secondary structural motifs. iDeepS, BERT-RBP, and GraphProt2 are based on DNNs and are capable of outputting primary sequence motifs or linear structure profiles. GraphProt is the predecessor of GraphProt2, which is based on support vector machine. Infernal is a widely used program based on Profile CFG model. However, to build an initial Profile CFG model from a set of sequences, it requires multiple sequence alignment as input. The binding motifs of RNA-binding proteins are

often very short and have lengths of only a few bases. They are functionally conserved but not evolutionarily conserved across different transcript species. Further, the locations of the motif positions in the input sequences, which typically have length of a few hundred to thousands bases, are unknown. Therefore, it is difficult to construct a multiple alignment of peak regions in general. For this reason, Infernal is not included in the following computational experiments. Further comparison of our Profile CFG with Infernal is discussed in the supplementary section "Comparison with Infernal's Profile CFG".

2.4 Evaluation metric for accuracy of sequence-structure motif detection

In this study, we employed multiple metrics to assess the accuracy of motif detection from different perspectives. Among the metrics employed, one key metric is the sequence-wise AUROC, which measures discriminating accuracy between positive, negative, and decoy sequences. This evaluation of sequence-wise AUROC, based on a binding score computed for each sequence, is depicted in Figure 6 and Table 1 in the main text and Figure S10 and S3. While this metric is widely used in CLIP-Seq data analysis and offers significant advantages, especially when correct motifs are unknown, it measures the overall binding prediction accuracy, which can be influenced by global biases or features unrelated to the motif. Therefore, relying solely on this metric for motif discovery accuracy is considered inadequate.

To address this limitation, we also compared the AUROC in tasks that distinguish between motif and background regions within a positive sequence. The position-wise AUROC, another vital metric, utilizes binding scores generated by tools for each position in the sequence. This approach is effective in verifying whether the model precisely identifies motif regions within sequences. However, it necessitates prior knowledge of the correct motif positions and a tool's capability of outputting signal strengths at each position. Table 2 in the main text demonstrates the comparison using this metric.

Furthermore, RNAelem can output not only the signal strength at each position, but also the most likely estimated (MLE) secondary structure within the motif region determined by the Viterbi algorithm. This feature enables a direct evaluation of the congruence between the learned motifs and the correct motifs in terms of structure, as assessed using the Matthews Correlation Coefficient (MCC) in Figure S6.

By integrating these evaluation results, we have demonstrated the superiority and precision of RNAelem in motif prediction. This highlights its efficacy in not only detecting motifs but also in aligning them structurally, thereby contributing significantly to the field of motif analysis in CLIP-Seq data.

3 Supplementary experiments

Next, we examined the relationships between information content of the primary sequence in the motif and the AUROC for the positive versus negative and positive versus decoy tasks (Figure S5). The information content of the primary sequence in the motif is varied by setting ambiguous IUPAC codes to the true motifs to generate positive sequences. RNAelem showed the greatest accuracy across all ranges of information content. For positive versus negative data, the accuracies of RNAelem, iDeepS, BERT-RBP, and GraphProt were not significantly changed or were only slightly increased with increasing information content. This suggests that these tools effectively utilized the structural information in addition to the sequence information. For positive versus decoy data, the accuracy of these tools declined slightly with increasing information content values, presumably because the decoy sequences also contained highly conserved sequences. The accuracy of CMFinder increased with increasing information content, suggesting that it is more sensitive to sequence conservation than to structural conservation. The accuracies of RNApromo and GraphProt2 decreased or showed nonmonotonic behavior, suggesting that they have limitations in utilizing sequence conservation efficiently.

We tested more rigorously the accuracy of the secondary structure of the motifs reported by RNAelem. The structure types were categorized more finely: base pairs, hairpin loops, bulge/in-

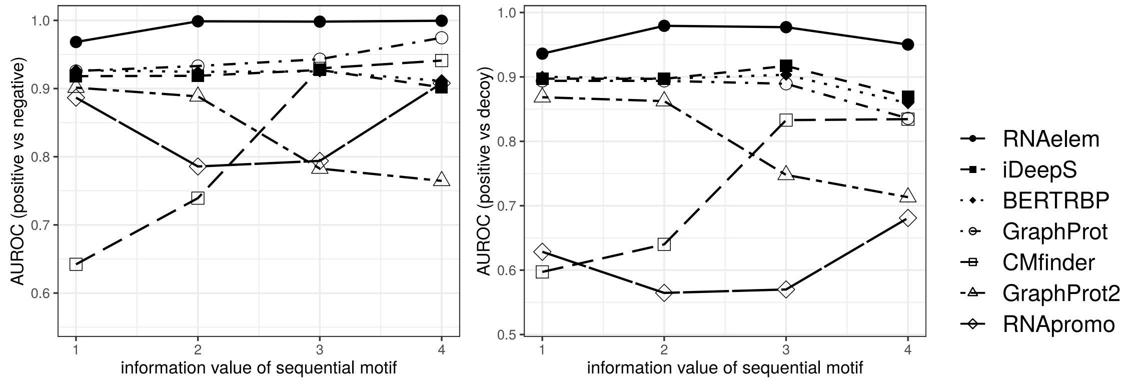

Supplementary Figure S5: **Dependency of AUROC on the information content of primary motifs.** The horizontal axis presents the Shannon information content of the primary sequence motif in the simulation data, and the vertical axis gives the AUROC for discriminating between positive vs negative sequences (left panel) or positive vs decoy sequences (right panel).

ternal/multifurcation loops, outer loops. Using RNAelem's maximum likelihood parse tree, the secondary structures of the motifs aligned with each sequence were predicted. Agreement between the predicted and ground truth (GT) secondary structure was examined using the Mathews Correlation Coefficient (MCC) (Figure S6). Increasing the maximum span W of RNAelem generally improved the accuracy of every type of structure, with the exception of base pairs. This may be because it becomes more difficult to predict base pairs with increasing maximal span as the number of base pair combinations is proportional to the square of the distance. The hairpin loop showed the highest accuracy for all loop types. Interestingly, the estimation of insertion regions was as accurate as base pairs and loops when the maximum span was 50 or greater.

Additionally, it was confirmed that our model selection approach using Enrichment Score (ES) effectively mitigates the risk of false positive motif detection. A control dataset, created by substituting positive sequences with 100 new negative sequences, was used to train a negative control model. The ES histogram for each pattern, as shown in the Figure S7, displays a sharp peak with a mean of 0.482 and a standard deviation of 0.0414, signifying the limited discriminatory capability of the trained models. For the shortest pattern "*...*", the ES was 0.464, underscoring its expected insignificance.

3.1 Enrichment Scores for ground truth search patterns

In this study, the search patterns employed comprehensively encompass possible secondary structures of length 10, which means that the secondary structures of the ground truth (GT) motifs embedded in the simulation datasets are also included. We define the pattern corresponding to the GT motif's secondary structure as the "GT search pattern." The presence of GT search patterns implies a potential bias, suggesting that RNAelem may unfairly yield favorable results by "knowing" the correct motif structures in advance. To address this concern, we investigated the impact of GT search patterns on RNAelem's outcomes.

We introduce the "GT-masked search pattern set," a specific subset of search patterns that excludes both the GT motifs and patterns with an insertion region at the center of the GT. This is illustrated in Figure S8, which compares the Enrichment Scores (ES) for search patterns selected from three different candidate pools: the complete search pattern set, the GT-masked set, and the GT pattern alone. An analysis of 43 simulated datasets revealed that, except for one dataset, the ES for the selected search patterns remained consistent across datasets. Interestingly, the ES for the GT search patterns were consistently lower than those of the selected patterns.

This result can be attributed to the overlap in the expressive capabilities of the search patterns. Drawing inspiration from RNAshapes (Steffen et al., 2006), our patterns use an abstract structure

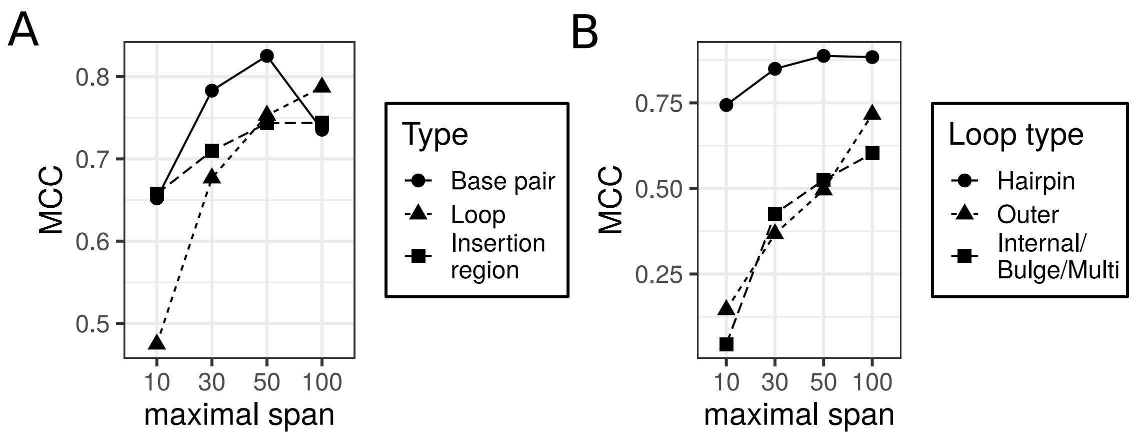

Supplementary Figure S6: **Accuracy of RNAelem's maximum likelihood estimation (MLE) for the secondary structure of the motif region.** The horizontal axis represents the maximum distance between base pairs allowed by the model (maximal span), and the vertical axis gives the Matthews correlation coefficient (MCC) that is plotted for each type of secondary structure. **(A)** Base pairs, loops, and insertion regions. **(B)** Subdivided loop region categories: hairpin, outer, and internal/bulge/multi loops.

notation, increasing pattern matching flexibility. Thus, different patterns can represent overlapping secondary structures, meaning the GT motif structure is not exclusively covered by the GT search patterns. Moreover, the common subsequences and substructures of embedded GT motifs are localized within specific positions in the motif. Therefore, the most fitting search patterns may differ from the GT motifs. We suggest that search patterns tailored to these localities in GT motifs gained more significance.

Figure S10 presents the ES for each of 167 RBPs, grouped based on two aspects: (1) whether the best motif contains a stem region, and (2) whether it contains an insertion region. The data suggests that motifs containing base-pairs are likely to contain insertion regions, with corresponding high ESs, implying a binding configuration akin to Figure 1 occurs frequently. Conversely, loop-only motifs are less likely to contain insertion regions, potentially indicating that conserved base-pairing is necessary for an RBP to stably bind to separate parts of a target RNA.

The motifs with the top ESs are depicted in Figure S11. Figure S11A lists the RBPs whose top three motif models exhibit a loop structure. Searching for sequence motifs in single-stranded regions is relatively simple and could be performed by previous tools, such as the tools referenced in Ref. (Hiller et al., 2006), which search for primary sequence motifs in structurally accessible regions. Our results are in alignment with previous studies (Hiller et al., 2006; Gao et al., 1994; Hafner et al., 2010). Additionally, Figure S11B presents RBPs that contain stem structures in optimal or suboptimal motifs, which potentially provide new insights.

3.2 Ensemble learning using suboptimal motifs

As our primary motivation is to pinpoint structural motifs that the RBP directly contacts or recognizes, using multiple suboptimal motifs to improve the accuracy for discriminating between positive and negative sequences is slightly out of our scope. However, we did preliminary experiments to investigate whether suboptimal motifs have additional useful information in this revision.

We simply used the binding strengths output by all motif models as 135-dimensional explanatory variables and trained a binary classification task to predict the presence or absence of binding using the gradient boosting machine (GBM) CatBoost. The learning parameters were set with a depth of 10 and 1000 optimization iterations. Figure S12A shows that the AUROC of RNAelem with CatBoost on eCLIP data reached 0.867, ranking second to BERT-RBP (0.882). Given that the features

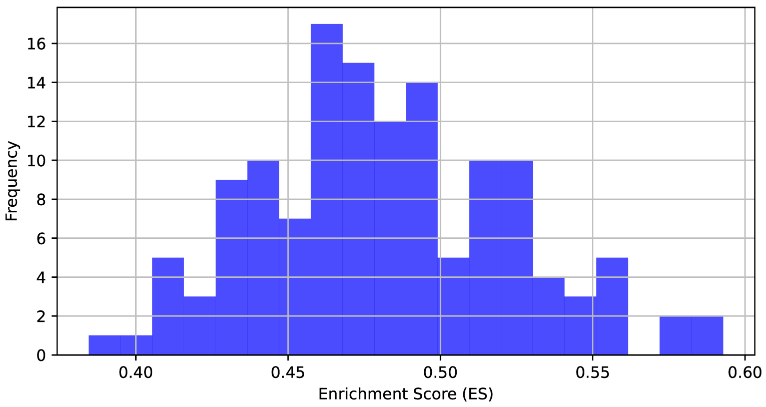

Supplementary Figure S7: **Histogram of Enrichment Score (ES) for different motif search patterns using a negative control model.** Each value in the histogram corresponds to the ES for a specific search pattern, indicating the model's ability to discriminate between positive and negative sequences.

are solely based on sequence-structure motifs, their significant contribution to determining RBP-RNA interactions is clearly demonstrated. Interestingly, the ES of suboptimal motifs did not always correlate with the feature importance learned by CatBoost (correlation coefficient 0.0641). While some suboptimal motifs exhibited high feature importance, this suggests that complex interactions may play a crucial role (Figure S12B).

Comparison of computational time

We compared the processing times for training and testing using both simulated and eCLIP data (Table S5). The processing environment consisted of an Intel(R) Core(TM) i9-9900KF CPU @ 3.60GHz and an NVIDIA GeForce RTX 2070 SUPER. However, as RNAelem requires parallel computing on a cluster machine, it was not possible to measure using the entire dataset on this benchmark machine. Therefore, we reduced the number of iterations for parameter optimization to five for ten datasets and recorded the execution. We then compared this with records from computations on a cluster machine for the same datasets, calculating the fold increase in processing time per iteration. Finally, we estimated the processing times for handling the datasets on the benchmark machine. These estimated values are marked with an asterisk in the table.

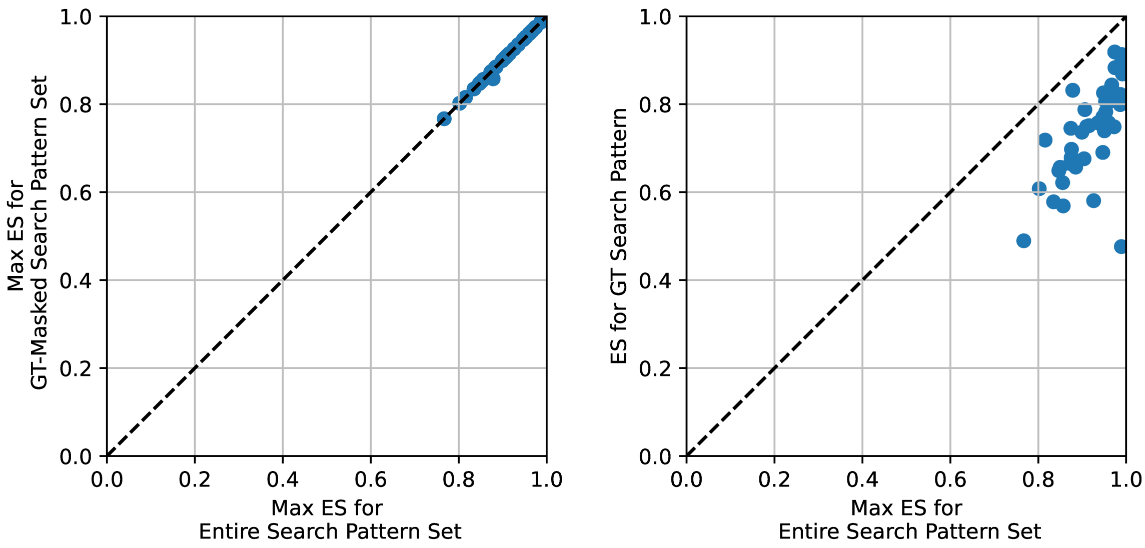

Supplementary Figure S8: **Comparative analysis of Enrichment Score (ES) across simulated datasets.** This figure depicts the ES for selected search patterns, evaluated by model selections across different candidate pools: the complete search pattern set, the GT-masked search pattern set, and the GT patterns alone.

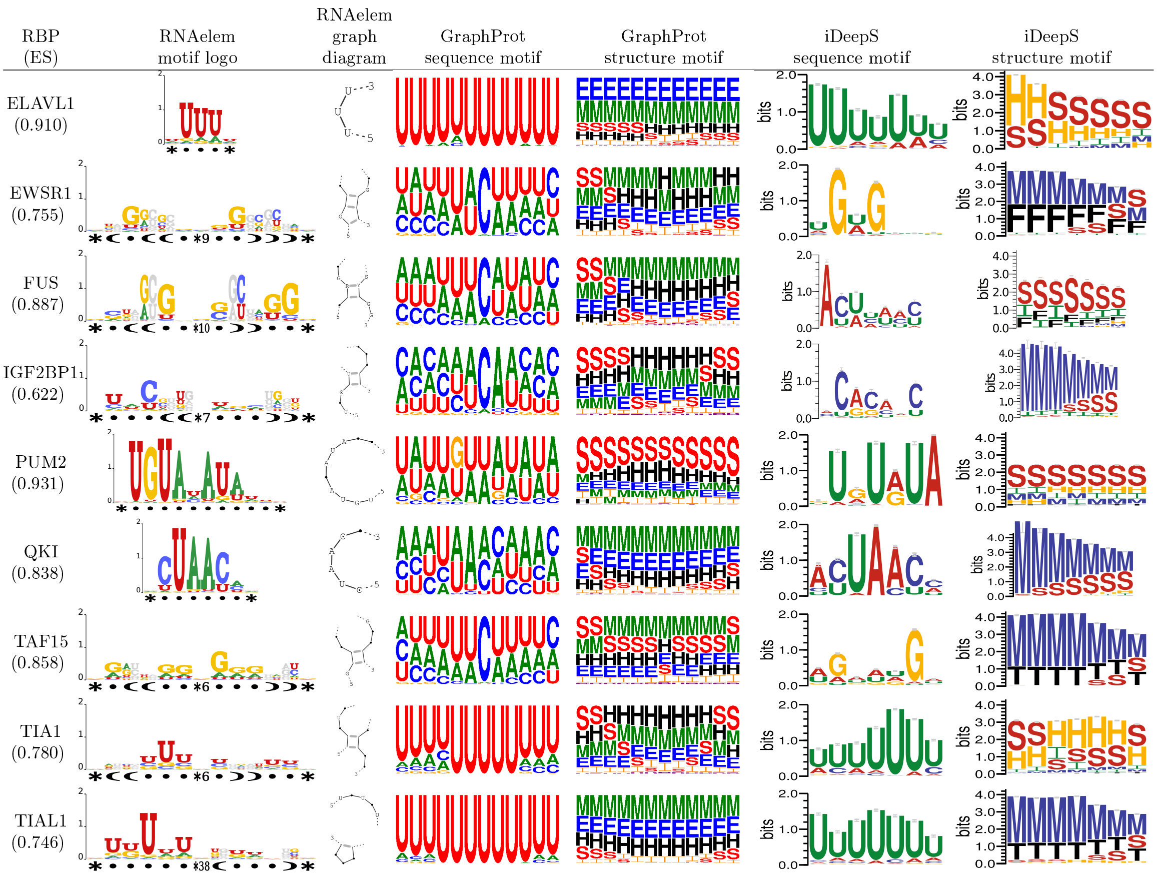

Supplementary Figure S9: **Comparison of motif visualizations among motif search tools** The figure illustrates motif logos generated by tools designed for outputting binding sequence-structure motifs of RNA-binding proteins (RBPs). For GraphProt (Maticzka et al., 2014) and iDeepS (Pan et al., 2018), the motif logos have been retrieved from their original publications.

Supplementary Table S2: **List of structural motifs embedded in the simulated data.** The information content of motif sequences was calculated from the alignment of the dataset. Structural stability was calculated with the Vienna RNA package (Lorenz et al., 2011) from base pair probabilities of motif regions and loop probabilities of loop regions.

	Motif structure in RNA secondary structure types	Motif structure in dot-bracket format	Motif sequence in IUPAC ambiguous bases	Information of motif sequences in dataset	Structural stability
1	IIILLHHHRR	...((...))	ACSGSYGAGC	18.5	0.67
2	ILHHHHHHHR	.(.......)	KCMSUKYWWG	14.6	0.63
3	ILHHHHHHHR	.(.......)	MCAWWUGCSG	17.1	0.56
4	ILHHHHHHHR	.(.......)	GCAAACACCG	20.0	0.66
5	ILHHHHHHHR	.(.......)	KNMWNNNNVN	8.16	0.63
6	ILHHHHHHHR	.(.......)	HBANRDBSHC	11.4	0.57
7	ILLHHHHRRI	.((....)).	USHYYWUGMM	17.2	0.96
8	ILLHHHHRRI	.((....)).	MCCGUGAGGR	18.4	0.89
9	ILLHHHHRRI	.((....)).	UGAAACAUCU	20.0	0.72
10	ILLHHHHRRI	.((....)).	NBNRBNHYNH	7.36	0.75
11	ILLHHHHRRI	.((....)).	HBRGKDNCVN	11.2	0.88
12	IIILLHHHRR	...((...))	NDKNCNNNBN	8.57	0.67
13	LBLHHHHHRR	(.(.....))	VVNNNCVDNN	8.97	0.56
14	LHHHHHRIII	(.....)...	MAAYKUGWAG	17.5	0.52
15	LHHHHHRIII	(.....)...	CAACUUGAAG	20.0	0.52
16	LHHHR*HHHHH	(...)*.....	CACAG*UAAAU	20.0	0.70
17	LHHHR*IIIII	(...)*.....	CACAG*UAAAU	20.0	0.70
18	LHHHR*IIIII	(...)*.....	BNNNN*BNNCM	6.63	0.69
19	LHHHR*MMMMM	(...)*.....	CACAG*UAAAU	20.0	0.72
20	LHHHR*MMMMM	(...)*.....	BNNNN*BNNCM	6.78	0.65
21	LHHHR*OOOOO	(...)*.....	CACAG*UAAAU	20.0	0.74
22	IIILLHHHRR	...((...))	GMABDDBNNY	12.6	0.59
23	LHHHR*OOOOO	(...)*.....	SSSRD*BBSYD	14.8	0.70
24	LLHHHHHHRR	((......))	SKWMWBRAYM	15.8	0.99
25	LLHHHHHHRR	((......))	CRKURAUUUG	18.5	0.95
26	LLHHHHHHRR	((......))	NWNAHNHNNV	6.27	0.90
27	LLHHHHHHRR	((......))	DRBKUVDBSV	12.3	0.94
28	LLHHHRRIII	((...))...	GGUUCCCUGU	20.0	0.60
29	LLHHHRRIII	((...))...	VVUDNNNNNH	9.52	0.68
30	LLHHHRRIII	((...))...	VNDWGSDKWD	12.3	0.56
31	MLLHHHHRRM	.((....)).	MCCGUGAGGR	19.0	0.75
32	MMMLLHHHRR	...((...))	ACSGSYGAGC	19.4	0.57
33	IILHHHHHHR	..(......)	KWDRGUWWCM	16.9	0.60
34	MMMLLHHHRR	...((...))	NDKNCNNNBN	11.1	0.56
35	OLLHHHHRRO	.((....)).	MCCGUGAGGR	18.7	0.69
36	OLLHHHHRRO	.((....)).	HBRGKDNCVN	12.7	0.70
37	OOOLLHHHRR	...((...))	NDKNCNNNBN	12.0	0.52
38	IILHHHHHHR	..(......)	GAGCUUYSRC	18.0	0.61
39	IILHHHHHHR	..(......)	ACCGAUAUAG	20.0	0.61
40	IILHHHHHHR	..(......)	HNNNNAWNNN	6.90	0.56
41	IILHHHHHRI	..(.....).	WKCRRSWDSA	14.9	0.62
42	IILHHHHHRI	..(.....).	UMCUCWSAGC	17.8	0.51

Supplementary Table S3: **AUROC comparison across simulated and eCLIP data for various tools.** Evaluation of mean AUROC values for 43 simulated datasets and 167 eCLIP datasets.

	Main Algorithm	Simulated Data		eCLIP Data
		positive vs. negative	positive vs. decoy	
CMfinder	CFG	0.864	0.769	0.585
RNApromo	CFG	0.861	0.632	0.621
GraphProt	SVM	0.954	0.865	0.846
iDeepS	DNN	0.912	0.888	0.848
GraphProt2	DNN	0.808	0.766	—
BERT-RBP	DNN	0.919	0.880	**0.882**
RNAelem	CFG	**0.995**	**0.959**	0.726

Supplementary Table S4: **AUROC comparison for motif position estimation.** AUROC was calculated separately for the entire motif region, stem region, and loop region.

	Entire region	Stem region	Loop region
CMfinder	0.729	0.722	0.728
RNApromo	0.795	0.856	0.763
GraphProt	0.765	0.667	0.803
GraphProt2	0.630	0.627	0.629
RNAelem	**0.987**	**0.979**	**0.981**

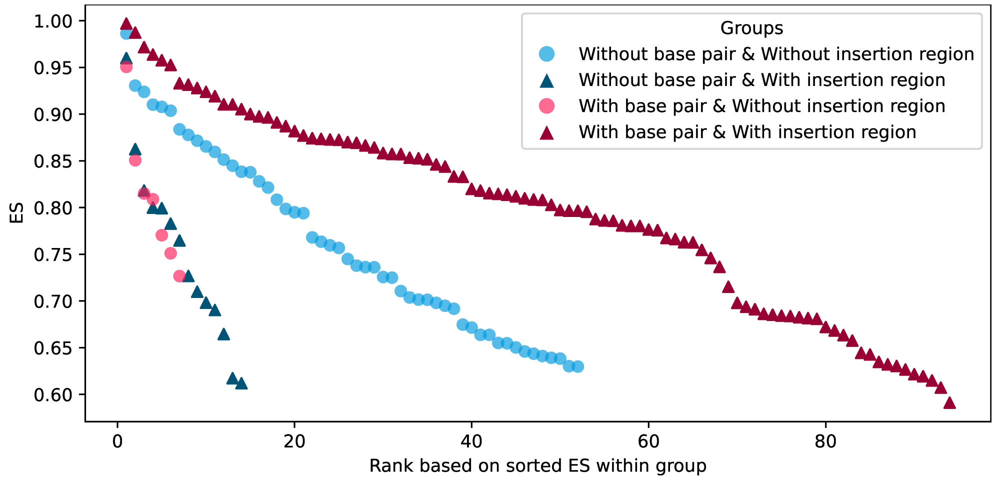

Supplementary Figure S10: **ES of the predicted sequence-structure motifs for eCLIP data.** Each point corresponds to an RBP for which there is CLIP-seq data in the eCLIP database. The vertical axis presents the ES for discriminating between binding and non-binding regions, and the horizontal axis represents the ranking order of ES. The RBPs are grouped based on two aspects: (1) whether the best motif contains a stem region, and (2) whether the motif contains an insertion region.

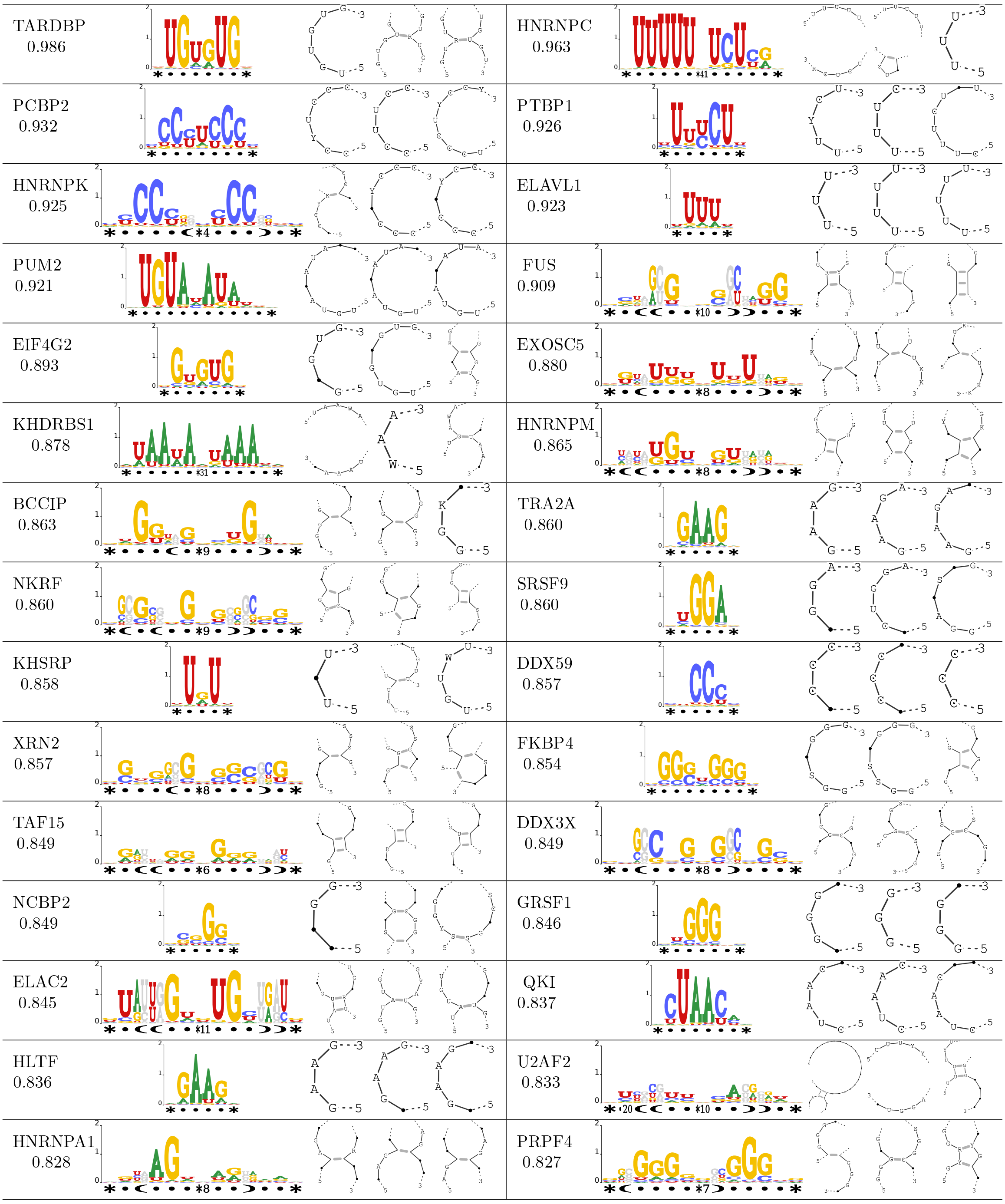

Supplementary Figure S11: **List of structural motifs with high ES detected from the eCLIP data.** The names of the RBPs, ESs, sequence logos for optimal motifs, and graph diagrams for suboptimal motifs are displayed.

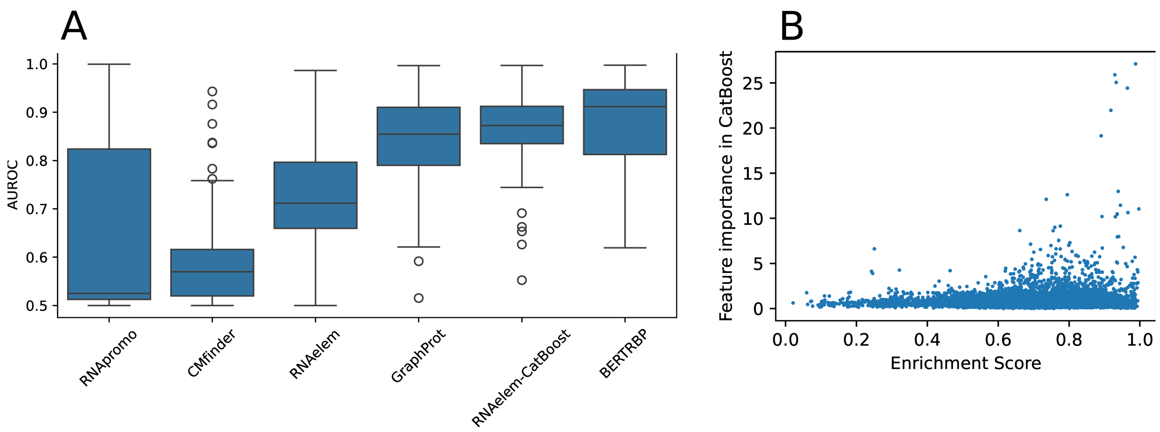

Supplementary Figure S12: **Ensemble learning using suboptimal motifs.** **A** Comparison including ensemble learning for binding strength prediction in eCLIP data. By training CatBoost with suboptimal motifs from RNAelem as explanatory variables, we predict binding strength in eCLIP data and calculate the AUROC (RNAelem-CatBoost). **B** The relationship between ES reported by RNAelem and feature importance reported by RNAelem-CatBoost is shown for all suboptimal motifs across all eCLIP data.

Supplementary Table S5: **Computational time of each tool for training and testing.** Average time for each dataset in both simulation and eCLIP scenarios. A value with an asterisk indicates an estimated value when run on the same benchmark machine as others.

**Model**	**Simulation**		**eCLIP**	
	**Training** [s] ($\frac{N=200}{positive,negative}$)	**Test** [s] (N=300)	**Training**[s] ($\frac{N=2000}{positive,negative}$)	**Test**[s] (N=1000)
CMfinder	8.41	2.76	67.3	6.06
RNApromo	1.95	1.01	12.7	4.01
GraphProt	9.03	65.2	47.5	227
iDeepS	23.4	1.34	79.5	3.68
GraphProt2	19.0	21.0	-	-
BERT-RBP	30.9	1.22	55.4	3.53
RNAelem	1.89×10^5*	11.5	1.19×10^5*	19.1

(*) Estimated values

Supplementary Table S6: **Comparison with already predicted and experimentally validated motifs.** This table presents motifs from RNAelem with the highest ES, compared to those reported by GraphProt and iDeepS. It includes experimentally validated motifs and the RNA sequences used in corresponding RBP-RNA co-crystal structures in the PDB.

RBP	ES	RNAelem motif	GraphProt motif<br>- sequence<br>- structure	iDeepS motif<br>- sequence<br>- structure	Validated binding motif	Interacting RNA in PDB<br>(PDB ID)
ELAC2	0.997	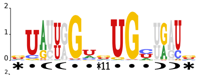	-	-	Acceptor stem of tRNA<br>(Meynier et al., 2024)	Mitochondrial precursor tRNA-His (8CBL)
TARDBP	0.986	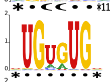	-	-	UG repeat<br>(Xiao et al., 2011)	-
PRPF4	0.972	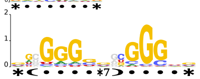	-	-	-	-
HNRNPC	0.960	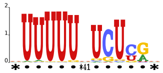	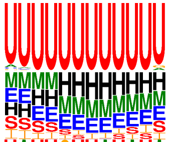	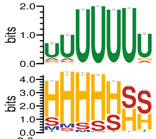	U-rich<br>(Cienikova et al., 2014)	-<br>AUUUUUC (2MXY)<br>UUUUC (2MZ1)
PUM2	0.931	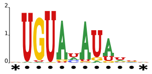	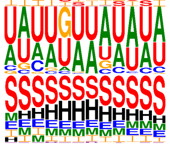	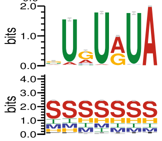	UGUANAUA<br>(Lu and Hall, 2011)	UGUAAAUA (3Q0Q)<br>UGUAGAUA (3Q0R)<br>UGUACAUC (3Q0S)
HNRNPK	0.928	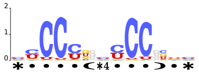	-	-	C-patch on hairpin<br>(Nakamoto et al., 2020)	-
ELAVL1	0.910	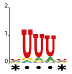	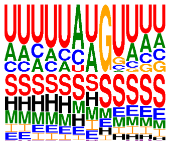	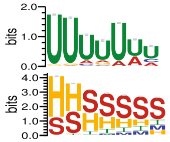	AU-rich<br>(Ripin et al., 2019; Pabis et al., 2019)	AUUUUUAUUUU (6GC5)<br>UAUUUA (6GD3)<br>UUUUUU (6G2K)<br>AUUUUUAUUUUAUUU (6GD2)
PTBP1	0.908	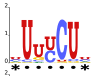	-	-	U-rich, Py-tract<br>(Oberstrass et al., 2005)	CUCUCU (2AD9, 2ADC)
PCBP2	0.904	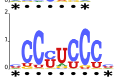	-	-	C-rich<br>(Du et al., 2007)	AACCCUAACCCU (2PY9)

RBP	ES	RNAelem motif	GraphProt motif<br>- sequence<br>- structure	iDeepS motif<br>- sequence<br>- structure	Validated binding motif	Interacting RNA in PDB<br>(PDB ID)
EXOSC5	0.891	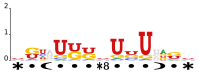	-	-	-	-
FUS	0.887	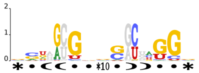	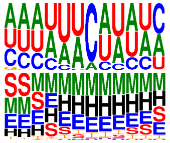	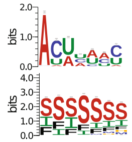	Variable length stem loop and downstream GGU<br>(Jutzi et al., 2020; Loughlin et al., 2019)	GGGAUUUCCCCAAAUGUGGAAACUCCC (6SNJ)<br>UGGUG (6G99)<br>GGCAGAUUACAAUUCUAUUUGCC (6GBM)
EIF4G2	0.884	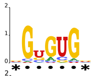	-	-	GUG<br>(Takahashi et al., 2005)	-
BCCIP	0.882	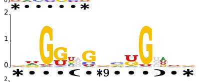	-	-	-	-
TRA2A	0.878	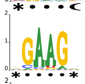	-	-	-	-
NKRF	0.877	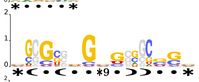	-	-	-	-
NCBP2	0.872	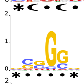	-	-	m7GpppG cap<br>(Dubiez et al., 2024)	GTG (8BY6)<br>MGT (8PMP, 8PNT)
HNRNPM	0.869	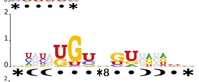	-	-	GU-rich<br>(Datar et al., 1993)	-
KHSRP	0.865	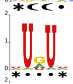	-	-	AU-rich<br>(Díaz-Moreno et al., 2010)	-
KHDRBS1	0.863	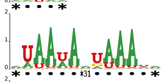	-	-	UAAA<br>(Chen et al., 1997)	-
FKBP4	0.860	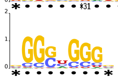	-	-	-	-
TAF15	0.858	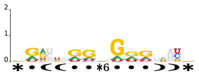	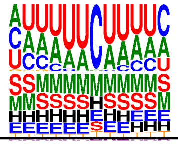	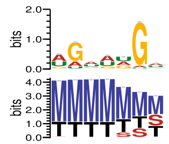	Stem loop, low sequence specificity<br>(Kashyap et al., 2015)	SON stem-loop RNA (2MMY), finetuned by MD

RBP	ES	RNAelem motif	GraphProt motif<br>- sequence<br>- structure	iDeepS motif<br>- sequence<br>- structure	Validated binding motif	Interacting RNA in PDB<br>(PDB ID)
U2AF2	0.857	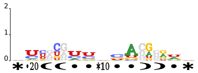	-	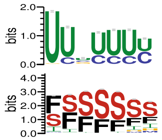	U-rich, Py-tract<br>(Maji et al., 2020)	UUDUUUBRUCC (6XLV, 6XLX, 6XLW)
XRN2	0.852	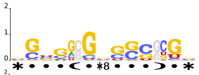	-	-	-	-
DDX59	0.851	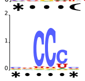	-	-	-	-
QKI	0.838	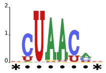	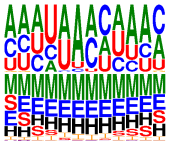	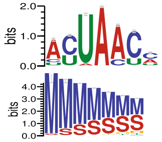	CUAAC<br>(Teplova et al., 2013)	UUCACUAACAA (4JVH)
SRSF9	0.838	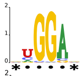	-	-	AGGAG<br>(Ha et al., 2021)	-
DDX3X	0.833	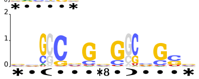	-	-	Double-stranded RNA<br>(Song and Ji, 2019)	CAAGGUCAUUCGCAAGAGUGGCCUUGCG<br>(6O5F, 8SSW)
HNRNPA1	0.812	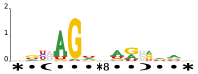	-	-	AGU on stem loop<br>(Morgan et al., 2015)<br>Two UAG on stem loop<br>(Kooshapur et al., 2018)<br>CAG...UAGG<br>(Beusch et al., 2017)	AGU (4YOE)<br>AGUAGAUUAGCA (6DCL)<br>UUAGGUC (5MPG)<br>UCAGUU (5MPL)
GRSF1	0.794	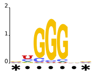	-	-	AGGGD<br>(Antonicka et al., 2013)<br>G-qudruplex<br>(Pietras et al., 2018)	AGGGUG (4QU6)<br>AGGGAU (4QU7)

Comparison with experimentally validated motifs

For ELAVL1, the RRM3 domain's recognition of accessible U residues was demonstrated through co-crystal structural analysis with sequences UUUUUU and UAUUUA (Pabis et al., 2019). RNAelem pinpointed 3-mer U motifs, which displayed a higher Enrichment Score compared to those in loops of length 4 to 10. GraphProt showed a preference for broader U-rich regions, whereas iDeepS favored stem areas. For FUS, which comprises RRM and ZnF domains, the RRM specifically recognizes YNY sequences at the 3' end of hairpin loops, and the ZnF domain binds to downstream GGU sequences (Loughlin et al., 2019). RNAelem identified similar motifs from eCLIP data, suggesting that Y residues at hairpin ends may form base pairs. This insight is not observable with other tools. For PUM2, its PUF domain specifically recognizes the single-stranded UGUANAUA sequences, a finding supported by co-crystal structures (Lu and Hall, 2011). Despite other tools reporting aligned findings due to sequence conservation, RNAelem's outputs remain distinctively accurate.

Limitations and capabilities regarding higher-order RNA structures

Our model, based on a context-free grammar, represents secondary structures and so cannot fundamentally account for higher-order structures that deviate from this norm. Therefore, the accuracy of motif estimation may decrease for RBPs where these higher-order structures are crucial for binding region recognition. However, currently available data is scarce, making it difficult to investigate the effects of higher-order structures on the accuracy of our motif-finding method.

Regarding coaxial stacking, the Turner energy model we use includes simple coaxial stacking. For example, in secondary structures like "((((*)).*.))" or "((.((*)).*..))", the energy stabilization effect of coaxial stacking in internal or multi-loops is included in the energy parameters. Currently, there is no eCLIP data for RBPs where coaxial stacking is significant for binding region recognition, but if such data becomes available, we might be able to evaluate the impact of coaxial stacking on motif estimation by excluding the effect of coaxial stacking from the energy parameters.

For other higher-order structures, GRSF1 is reported to bind to G-quadruplex structures (Pietras et al., 2018), and RNAelem outputs a GGG motif existing in loop regions (Table S6), consistently with the known fact. HNRNPF is also known to bind to G-quadruplex structures. However, there is no eCLIP data for HNRNPF yet. Regarding pseudoknots, since pseudoknots can be considered as interactions between distantly located stem-loops, RNAelem, which allows for long insertion regions, might be extended to estimate structural motifs and interaction mechanisms of RBPs recognizing pseudoknots.

4 Command line example

This example shows how to identify sequence-structure motifs in RNA sequences bound by RBPs using two FASTA files: `positive.fa` for positive sequences and `negative.fa` for negative sequences. If interested in hairpin structures with stem lengths of 3 and loop lengths of 3 to 5, search patterns of interest are specified in `pattern.txt` as follows:

```
$ cat pattern.txt
*(((...)))*
*(((...)))*
*(((....)))*
*(((.....)))*
```

To train the motif model, use the command:

```
$ elem pipeline -p positive.fa -n negative.fa -m pattern.txt
```

The training output is stored in the `elem_out` directory by default, with the optimal model in `elem_out/model-0`. Sub-optimal models are stored in `elem_out/model-1`, and so on. The optimal model directory contains:

```
$ ls elem_out/model-0
train.model prf.png rss.png
```

where `train.model` is the file containing motif model parameters for Coupled CFG, `prf.png` is the sequence logo, and `rss.png` is the graph diagram.

To search for the trained motifs in new sequences, run:

```
$ elem scan -s test.fa -m elem_out/model-0/train.model
```

Results are saved in `scan_out` by default. The format of search results is detailed as follows:

```
$ cat scan_out/raw.txt
id: @1
start: [-inf,-22.4701,-29.1701,-inf,-18.9275,-19.1997,-48.5456,-20.3391,...
end: [-inf,-inf,-inf,-inf,-inf,-inf,-inf,-inf,-inf,-inf,-inf,-inf,-inf,-inf,...
inner: [-inf,-22.4701,-22.4689,-22.4689,-18.899,-18.3449,-18.3449,-18.2173,...
psihat: [0,0,0,0,0,0,0,0,0,0,0,0,0,0,0,0,0,0,0,0,0,0,0,0,0,0,0,0,0,0,0,0,0,0,...
motif region: 51 - 64
exist prob: 0.00518753
seq: GCAUCCAUGGCUGAAUGGUUAAAGCGCCCAACUCAUAAUUGGUAAAUUUGCGGGUUCAAUUCCUGCUGGAUGCA
rss: OOOOLLLLHHHHHHRRRROOOOOLLLILLLLLBLLHHHHRRRRMMMMMMLLLLLHHHHHHHRRRRRRRIRRRO
mot:                                                   (((.......)))
(Repeat for each sequence)
```

where each line providing specific types of information:

- **id:** The sequence identifier.
- **start:** A list of log-scaled probabilities indicating the likelihood of the motif's start position at each sequence position.
- **end:** A list of log-scaled probabilities for the motif's end positions, similar to start.
- **inner:** Log-scaled probabilities for each position being inside the motif.
- **psihat:** Represents the best alignment of the Profile CFG with each number indicating the index in the search pattern, starting from 0 for the first non-insertion region.
- **motif region:** Indicates the most likely span of the motif within the sequence.
- **exist prob:** The probability that the motif exists in the sequence.
- **seq:** The input RNA sequence.
- **rss:** The most likely RNA secondary structure corresponding to the best motif alignment.
- **mot:** Shows the alignment of the best motif match within the sequence.

This structure provides a comprehensive approach to motif discovery in RNA sequences.

References

Antonicka, H., Sasarman, F., Nishimura, T., Paupe, V., and Shoubridge, E. A. (2013). The mitochondrial RNA-binding protein GRSF1 localizes to RNA granules and is required for posttranscriptional mitochondrial gene expression. *Cell metabolism*, 17(3):386–398.

Bailey, T. L., Boden, M., Buske, F. A., Frith, M., Grant, C. E., Clementi, L., Ren, J., Li, W. W., and Noble, W. S. (2009). MEME SUITE: tools for motif discovery and searching. *Nucleic acids research*, 37(suppl_2):W202–W208.

Beusch, I., Barraud, P., Moursy, A., Clery, A., and Allain, F. H.-T. (2017). Tandem hnRNP A1 RNA recognition motifs act in concert to repress the splicing of survival motor neuron exon 7. *Elife*, 6:e25736.

Chen, T., Damaj, B. B., Herrera, C., Lasko, P., and Richard, S. (1997). Self-association of the single-KH-domain family members Sam68, GRP33, GLD-1, and Qk1: role of the KH domain. *Molecular and cellular biology*.

Cienikova, Z., Damberger, F. F., Hall, J., Allain, F. H.-T., and Maris, C. (2014). Structural and mechanistic insights into poly (uridine) tract recognition by the hnRNP C RNA recognition motif. *Journal of the American Chemical Society*, 136(41):14536–14544.

Datar, K. V., Dreyfuss, G., and Swanson, M. S. (1993). The human hnRNP M proteins: identification of a methionine/arginine-rich repeat motif in ribonucleoproteins. *Nucleic acids research*, 21(3):439–446.

Díaz-Moreno, I., Hollingworth, D., Kelly, G., Martin, S., García-Mayoral, M., Briata, P., Gherzi, R., and Ramos, A. (2010). Orientation of the central domains of KSRP and its implications for the interaction with the RNA targets. *Nucleic acids research*, 38(15):5193–5205.

Du, Z., Lee, J. K., Fenn, S., Tjhen, R., Stroud, R. M., and James, T. L. (2007). X-ray crystallographic and NMR studies of protein–protein and protein–nucleic acid interactions involving the KH domains from human poly (C)-binding protein-2. *Rna*, 13(7):1043–1051.

Dubiez, E., Pellegrini, E., Brask, M. F., Garland, W., Foucher, A.-E., Huard, K., Jensen, T. H., Cusack, S., and Kadlec, J. (2024). Structural basis for competitive binding of productive and degradative co-transcriptional effectors to the nuclear cap-binding complex. *Cell Reports*, 43(1).

Eddy, S. R. (2002). A memory-efficient dynamic programming algorithm for optimal alignment of a sequence to an RNA secondary structure. *BMC bioinformatics*, 3:1–16.

Fang, X.-Y., Luo, Z.-G., and Wang, Z.-H. (2008). Predicting RNA secondary structure using profile stochastic context-free grammars and phylogenic analysis. *Journal of computer science and technology*, 23(4):582–589.

Gao, F.-B., Carson, C. C., Levine, T., and Keene, J. D. (1994). Selection of a subset of mRNAs from combinatorial 3'untranslated region libraries using neuronal RNA-binding protein Hel-N1. *Proceedings of the National Academy of Sciences of the United States of America*, 91(23):11207–11211.

Ha, J., Jang, H., Choi, N., Oh, J., Min, C., Pradella, D., Jung, D.-W., Williams, D. R., Park, D., Ghigna, C., et al. (2021). SRSF9 regulates cassette exon splicing of caspase-2 by interacting with its downstream exon. *Cells*, 10(3):679.

Hafner, M., Landthaler, M., Burger, L., Khorshid, M., Hausser, J., Berninger, P., Rothballer, A., Ascano Jr, M., Jungkamp, A.-C., Munschauer, M., et al. (2010). Transcriptome-wide identification of RNA-binding protein and microRNA target sites by PAR-CLIP. *Cell*, 141(1):129–141.

Hiller, M., Pudimat, R., Busch, A., and Backofen, R. (2006). Using RNA secondary structures to guide sequence motif finding towards single-stranded regions. *Nucleic acids research*, 34(17):e117–e117.

Jiang, M., Anderson, J., Gillespie, J., and Mayne, M. (2008). uShuffle: a useful tool for shuffling biological sequences while preserving the k-let counts. *BMC bioinformatics*, 9:1–11.

Jutzi, D., Campagne, S., Schmidt, R., Reber, S., Mechtersheimer, J., Gypas, F., Schweingruber, C., Colombo, M., von Schroetter, C., Loughlin, F. E., et al. (2020). Aberrant interaction of FUS with the U1 snRNA provides a molecular mechanism of FUS induced amyotrophic lateral sclerosis. *Nature Communications*, 11(1):6341.

Kashyap, M., Ganguly, A. K., and Bhavesh, N. S. (2015). Structural delineation of stem-loop RNA binding by human TAF15 protein. *Scientific reports*, 5(1):17298.

Kingma, D. P. and Ba, J. (2014). Adam: A method for stochastic optimization. *arXiv preprint arXiv:1412.6980*.

Kiryu, H., Kin, T., and Asai, K. (2008). Rfold: an exact algorithm for computing local base pairing probabilities. *Bioinformatics*, 24(3):367–373.

Kleinkauf, R., Mann, M., and Backofen, R. (2015). antaRNA: ant colony-based RNA sequence design. *Bioinformatics*, 31(19):3114–3121.

Kooshapur, H., Choudhury, N. R., Simon, B., Mühlbauer, M., Jussupow, A., Fernandez, N., Jones, A. N., Dallmann, A., Gabel, F., Camilloni, C., et al. (2018). Structural basis for terminal loop recognition and stimulation of pri-miRNA-18a processing by hnRNP A1. *Nature communications*, 9(1):2479.

Lorenz, R., Bernhart, S. H., Höner zu Siederdissen, C., Tafer, H., Flamm, C., Stadler, P. F., and Hofacker, I. L. (2011). ViennaRNA Package 2.0. *Algorithms for Molecular Biology*, 6.

Loughlin, F. E., Lukavsky, P. J., Kazeeva, T., Reber, S., Hock, E.-M., Colombo, M., Von Schroetter, C., Pauli, P., Cléry, A., Mühlemann, O., et al. (2019). The solution structure of FUS bound to RNA reveals a bipartite mode of RNA recognition with both sequence and shape specificity. *Molecular cell*, 73(3):490–504.

Lu, G. and Hall, T. M. T. (2011). Alternate modes of cognate RNA recognition by human PUMILIO proteins. *Structure*, 19(3):361–367.

Maji, D., Glasser, E., Henderson, S., Galardi, J., Pulvino, M. J., Jenkins, J. L., and Kielkopf, C. L. (2020). Representative cancer-associated U2AF2 mutations alter RNA interactions and splicing. *Journal of Biological Chemistry*, 295(50):17148–17157.

Mathews, D. H., Sabina, J., Zuker, M., and Turner, D. H. (1999). Expanded sequence dependence of thermodynamic parameters improves prediction of RNA secondary structure. *Journal of molecular biology*, 288(5):911–940.

Maticzka, D., Lange, S. J., Costa, F., and Backofen, R. (2014). GraphProt: modeling binding preferences of RNA-binding proteins. *Genome biology*, 15(1):1–18.

Meynier, V., Hardwick, S. W., Catala, M., Roske, J. J., Oerum, S., Chirgadze, D. Y., Barraud, P., Yue, W. W., Luisi, B. F., and Tisné, C. (2024). Structural basis for human mitochondrial tRNA maturation. *Nature Communications*, 15(1):4683.

Morgan, C. E., Meagher, J. L., Levengood, J. D., Delproposto, J., Rollins, C., Stuckey, J. A., and Tolbert, B. S. (2015). The first crystal structure of the UP1 domain of hnRNP A1 bound to RNA reveals a new look for an old RNA binding protein. *Journal of molecular biology*, 427(20):3241–3257.

Nakamoto, M. Y., Lammer, N. C., Batey, R. T., and Wuttke, D. S. (2020). hnRNPK recognition of the B motif of Xist and other biological RNAs. *Nucleic acids research*, 48(16):9320–9335.

Nawrocki, E. P. and Eddy, S. R. (2013). Infernal 1.1: 100-fold faster RNA homology searches. *Bioinformatics*, 29(22):2933–2935.

Oberstrass, F. C., Auweter, S. D., Erat, M., Hargous, Y., Henning, A., Wenter, P., Reymond, L., Amir-Ahmady, B., Pitsch, S., Black, D. L., et al. (2005). Structure of PTB bound to RNA: specific binding and implications for splicing regulation. *Science*, 309(5743):2054–2057.

Pabis, M., Popowicz, G. M., Stehle, R., Fernández-Ramos, D., Asami, S., Warner, L., García-Mauriño, S. M., Schlundt, A., Martínez-Chantar, M. L., Díaz-Moreno, I., et al. (2019). HuR biological function involves RRM3-mediated dimerization and RNA binding by all three RRMs. *Nucleic acids research*, 47(2):1011–1029.

Pan, X., Rijnbeek, P., Yan, J., and Shen, H.-B. (2018). Prediction of RNA-protein sequence and structure binding preferences using deep convolutional and recurrent neural networks. *BMC genomics*, 19(1):5–11.

Pietras, Z., Wojcik, M. A., Borowski, L. S., Szewczyk, M., Kulinski, T. M., Cysewski, D., Stepien, P. P., Dziembowski, A., and Szczesny, R. J. (2018). Dedicated surveillance mechanism controls G-quadruplex forming non-coding RNAs in human mitochondria. *Nature communications*, 9(1):2558.

Rabani, M., Kertesz, M., and Segal, E. (2008). Computational prediction of RNA structural motifs involved in posttranscriptional regulatory processes. *Proceedings of the National Academy of Sciences of the United States of America*, 105(39):14885–14890.

Ripin, N., Boudet, J., Duszczyk, M. M., Hinniger, A., Faller, M., Krepl, M., Gadi, A., Schneider, R. J., Šponer, J., Meisner-Kober, N. C., et al. (2019). Molecular basis for AU-rich element recognition and dimerization by the HuR C-terminal RRM. *Proceedings of the National Academy of Sciences*, 116(8):2935–2944.

Sankoff, D. (1985). Simultaneous solution of the RNA folding, alignment and protosequence problems. *SIAM journal on applied mathematics*, 45(5):810–825.

Song, H. and Ji, X. (2019). The mechanism of RNA duplex recognition and unwinding by DEAD-box helicase DDX3X. *Nature communications*, 10(1):3085.

Steffen, P., Voß, B., Rehmsmeier, M., Reeder, J., and Giegerich, R. (2006). RNAshapes: an integrated RNA analysis package based on abstract shapes. *Bioinformatics*, 22(4):500–503.

Takahashi, K., Maruyama, M., Tokuzawa, Y., Murakami, M., Oda, Y., Yoshikane, N., Makabe, K. W., Ichisaka, T., and Yamanaka, S. (2005). Evolutionarily conserved non-AUG translation initiation in NAT1/p97/DAP5 (EIF4G2). *Genomics*, 85(3):360–371.

Teplova, M., Hafner, M., Teplov, D., Essig, K., Tuschl, T., and Patel, D. J. (2013). Structure–function studies of STAR family Quaking proteins bound to their in vivo RNA target sites. *Genes & development*, 27(8):928–940.

Uhl, M., Heyl, F., Backofen, R., et al. (2021). GraphProt2: A graph neural network-based method for predicting binding sites of RNA-binding proteins. *BioRxiv*, page 850024.

Van Nostrand, E. L., Freese, P., Pratt, G. A., Wang, X., Wei, X., Xiao, R., Blue, S. M., Chen, J.-Y., Cody, N. A., Dominguez, D., et al. (2020). A large-scale binding and functional map of human RNA-binding proteins. *Nature*, 583(7818):711–719.

Van Nostrand, E. L., Pratt, G. A., Shishkin, A. A., Gelboin-Burkhart, C., Fang, M. Y., Sundararaman, B., Blue, S. M., Nguyen, T. B., Surka, C., Elkins, K., et al. (2016). Robust transcriptome-wide discovery of RNA-binding protein binding sites with enhanced CLIP (eCLIP). *Nature methods*, 13(6):508–514.

Xiao, S., Sanelli, T., Dib, S., Sheps, D., Findlater, J., Bilbao, J., Keith, J., Zinman, L., Rogaeva, E., and Robertson, J. (2011). RNA targets of TDP-43 identified by UV-CLIP are deregulated in ALS. *Molecular and Cellular Neuroscience*, 47(3):167–180.

Yamada, K. and Hamada, M. (2022). Prediction of RNA-protein interactions using a nucleotide language model. *Bioinformatics advances*, 2(1):vbac023.

Yao, Z., Weinberg, Z., and Ruzzo, W. L. (2006). CMfinder—a covariance model based RNA motif finding algorithm. *Bioinformatics*, 22(4):445–452.
